# Gap junction Delta-2b (*gjd2b*/Cx35.1) depletion causes hyperopia and visual-motor deficiencies in the zebrafish

**DOI:** 10.3389/fcell.2023.1150273

**Published:** 2023-03-02

**Authors:** Cherie A. Brown-Panton, Shiva Sabour, Georg S. O. Zoidl, Christiane Zoidl, Nima Tabatabaei, Georg R. Zoidl

**Affiliations:** ^1^ Department of Biology, York University, Toronto, ON, Canada; ^2^ Center for Vision Research, York University, Toronto, ON, Canada; ^3^ Department of Mechanical Engineering, York University, Toronto, ON, Canada; ^4^ Department of Psychology, York University, Toronto, ON, Canada

**Keywords:** electrical synapses, zebrafish, connexin, Cx36, eye, refractive error, behavior, optical coherence tomography

## Abstract

The zebrafish is a powerful model to investigate the developmental roles of electrical synapses because many signaling pathways that regulate the development of the nervous system are highly conserved from fish to humans. Here, we provide evidence linking the mammalian connexin-36 (Cx36) ortholog *gjd2b*/Cx35.1, a major component of electrical synapses in the zebrafish, with a refractive error in the context of morphological, molecular, and behavioral changes of zebrafish larvae. Two abnormalities were identified. The optical coherence tomography analysis of the adult retina confirmed changes to the refractive properties caused by eye axial length reduction, leading to hyperopic shifts. The *gjd2b*/Cx35.1 depletion was also correlated with morphological changes to the head and body ratios in larvae. The differential expression of Wnt/ß-catenin signaling genes, connexins, and dopamine receptors suggested a contribution to the observed phenotypic differences. The alteration of visual-motor behavioral responses to abrupt light transitions was aggravated in larvae, providing evidence that cone photoreceptor cell activity was enhanced when *gjd2b*/Cx35.1 was depleted. The visual disturbances were reversed under low light conditions in *gjd2b* ^*−/−*^/Cx35.1^−/−^ larvae. Since qRT-PCR data demonstrated that two rhodopsin genes were downregulated, we speculated that rod photoreceptor cells in *gjd2b*/Cx35.1^−/−^ larvae were less sensitive to bright light transitions, thus providing additional evidence that a cone-mediated process caused the VMR light-ON hyperactivity after losing Cx35.1 expression. Together, this study provides evidence for the role of *gjd2b*/Cx35.1 in the development of the visual system and visually guided behaviors.

## 1 Introduction

The transient coupling of groups of neurons by electrical synapses (gap junctions) is a phenomenon documented in different regions of the mammalian central nervous system across all life stages (Connors and Long, 2004; Connors, 2017; Pereda, 2019). This coupling is thought to be essential in several developmental events, including neuronal differentiation, cell death, cell migration, synaptogenesis, and neural circuit formation (Belousov and Fontes, 2013; Palacios-Prado et al., 2014; Haas et al., 2016; Pereda, 2016; Nagy et al., 2019). The formation of neuronal circuits and synchronized spontaneous activity are considered a hallmark of gap junction activity in the developing brain (Belousov and Fontes, 2013; Pereda, 2014).

At the molecular level, the principal component of mammalian neuronal gap junctions is connexin-36 (in mouse: *Gjd2*/Cx36) (Zoidl and Dermietzel, 2002; Connors and Long, 2004; Meier and Dermietzel, 2006). While the roles of Cx36 in the mature nervous system have been well established (Nagy et al., 2018; Nagy et al., 2019), less is known about the functions during mammalian development. It is thought that Cx36 serves canonical functions by coordinating metabolic and electrical activities in developing neurons and neuronal networks, but details of these roles remain to be resolved (Belousov and Fontes, 2013).

The zebrafish is an attractive model to investigate neurodevelopmental roles of electrical synapses since the signaling pathways that regulate brain development and function are highly conserved from fish to humans (Neuhauss, 2003; Wolman and Granato, 2012; Orger and de Polavieja, 2017; Gerlai, 2023). In teleost fish, like the zebrafish, several rounds of partial genome duplication have occurred (Postlethwait et al., 2004) that have increased the number of Cx36 orthologs; the ensuing evolutionary trajectories saw some connexin genes being duplicated and others lost (Eastman et al., 2006; Cruciani and Mikalsen, 2007; Zoidl et al., 2008). The *gjd2b/*Cx35.1 gene/protein (synonym: connexin 35b, Cx35b) belongs with *gjd1a/*Cx34.1, *gjd1b/*Cx34.7, and *gjd2a/*Cx35.5, two closely related subgroups of connexins with a shared homology of ≈85% between each other and mammalian connexin-36 (*Gjd2*/Cx36) (Eastman et al., 2006; Miller et al., 2017). Previous work has identified *gjd2b/*Cx35.1 in electrical synapses of the nervous system and the retina with minimal expression in other tissues (Zoidl et al., 2008; Li et al., 2009; Carlisle and Ribera, 2014). A high-resolution mRNA expression time course of embryonic development in zebrafish confirmed that *gjd2b/*Cx35.1 is expressed during neural induction, indicating a possible early role in neural progenitor cells (White et al., 2017). Additionally, *gjd2b/*Cx35.1 localizes to the ventral spinal cord during embryonic and early larval stages (Carlisle and Ribera, 2014). Further, a recent single-cell transcriptome analysis demonstrated *gjd2a/*Cx35.5 and *gjd2b/*Cx35.1 expression in a wide variety of retinal cell types of 2–5 days post fertilization (dpf) larval zebrafish (Quint et al., 2021).

The application of gene-editing tools in the zebrafish has allowed for new lines of investigation to provide insight into the diverse roles of Cx36 orthologs during development *via* loss-of-function studies. Research focusing on the Mauthner neural circuit, which mediates a fast escape behavior, demonstrated that the two Cx36 orthologs, *gjd1a/*Cx34.1 and *gjd2a/*Cx35.5, are required for synapse assembly and function at larval stages (Miller et al., 2017). The investigation also identified a genetic basis for molecular asymmetry at a vertebrate electrical synapse and showed that they are required for appropriate behavioral performance (Miller et al., 2017). Another study demonstrated that *gjd2b/*Cx35.1 mediated gap junctions regulate glutamatergic synapse formation and dendritic elaboration in Purkinje neurons of 5-8dpf larvae (Sitaraman et al., 2021). In a third study, depletion of *gjd2a/*Cx35.5 or *gjd2b/*Cx35.1 in zebrafish caused changes in the biometry and refractive status of the eye (Quint et al., 2021). The g*jd2a/*Cx35.5 depletion led to a hyperopic shift and electrophysiological changes in the retina, supporting a role for *gjd2a/*Cx35.5 in regulating ocular biometry. *Gjd2b*/Cx35.1 had an additional lenticular role, leading to a nuclear cataract that triggered axial elongation.

This study investigated the functional evidence linking *gjd2b*/Cx35.1 and refractive error in the context of morphological, molecular, and behavioral changes of 6-7dpf larvae when the visual response system is developed (Niell et al., 2004; Niell and Smith, 2005). We identified two abnormalities. An optical coherence tomography (OCT) analysis of the adult retina confirmed changes to the refractive properties caused by eye axial length reduction, leading to hyperopic shifts. Furthermore, *gjd2b*/Cx35.1 loss was correlated with morphological changes to the head and body ratios in larvae. Changes to Wnt/ß-catenin signaling, connexin (*gjd1b*/Cx34.7, *gja3*/Cx48.5), and dopamine receptor expression suggested a contribution to the observed phenotypic differences. The alteration of visual-motor behavioral responses to abrupt light transitions was aggravated, providing evidence that cone-cone photoreceptor cell activity was enhanced when *gjd2b*/Cx35.1 was lost. Visual disturbances were reversed under low/mesopic light conditions in *gjd2b* ^−/−^/Cx35.1^−/−^ larvae. Since qRT-PCR data demonstrated that rhodopsin genes were downregulated, we speculated that rod photoreceptor cells (PRCs) in *gjd2b* ^−/−^/Cx35.1^−/−^ larvae were less sensitive to bright light transitions, thus providing additional evidence that a cone-mediated process caused the VMR light-ON hyperactivity after losing Cx35.1 expression. Together, the main results of this study provide evidence for distinct roles of *gjd2b*/Cx35.1 in the development of the visual system affecting visually guided behaviors.

## 2 Materials and methods

### 2.1 Zebrafish lines and husbandry

Zebrafish (*Danio Rerio*) of the strain Tubingen long fin (TL) were maintained in groups with mixed sex in a recirculation system (Aquaneering Inc., San Diego, CA) at 28°C on a 14h light/10h dark cycle. All animal work was performed at York University’s zebrafish vivarium and in a S2 biosafety laboratory in accordance with the Canadian Council for Animal Care guidelines after approval of the study protocol by the York University Animal Care Committee (GZ#2019-7-R2). The Cx35.1 mutant line was generated and characterized in-house.

### 2.2 Generation of the *gjd2b* ^*−/−*^/Cx35.1^−/−^ line

**FIGURE 1:**
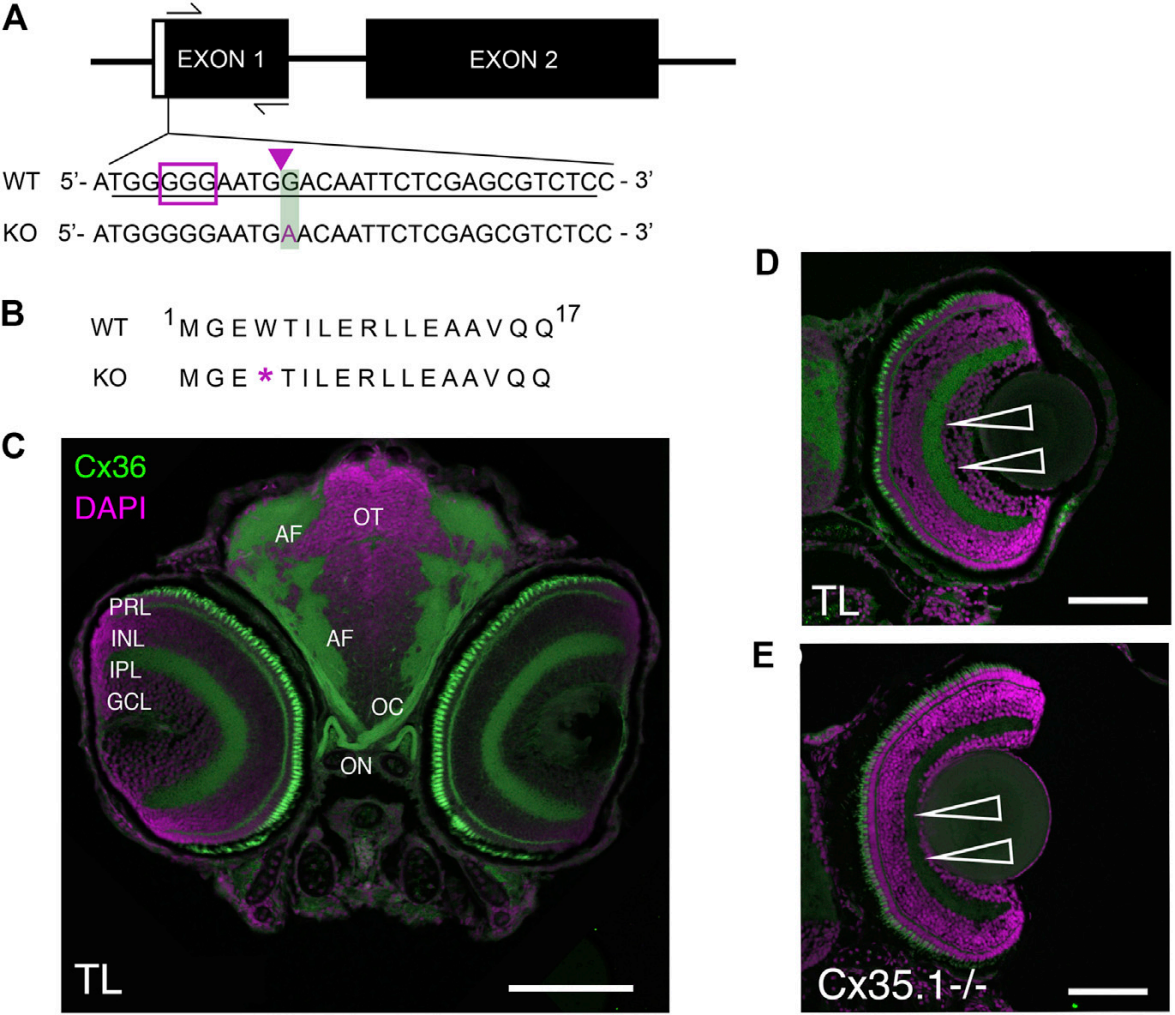
Generation and Primary Characterization of the *Gjd2b* ^*−/−*^ */*Cx35.1^−/−^ Line. **(A)** General overview of the *gjd2b* ^*−/−*^/Cx35.1^−/−^ gene structure and partial DNA sequence. The position of forward and reverse primers targeting exon one flanked the region containing CRISPR mutations. The sgRNA target with PAM sequence is outlined by the purple box; The predicted CRISPR-Cas9 binding region targeting a XhoI restriction site is indicated by the purple arrow. The CRISPR-Cas9 genome engineering strategy produced a 1bp substitution at position 12 of exon 1, resulting in the substitution of G to A in Cx35.1^−/−^ animals. The mutation is outlined with a green box. **(B)** Cx35.1 partial protein sequence. The mutation resulted in an early stop codon, as indicated by the purple asterisk (*). **(C)** Immunohistochemistry of *gjd2b* ^−/−^/Cx35.1^−/−^ larvae. (C1-left) A monoclonal anti-Cx36 primary antibody (Invitrogen) was applied to 6dpf wild type (TL) to determine Cx35 immunoreactivity in a frontal section of a zebrafish larva. The retina of *gjd2b* ^−/−^/Cx35.1^−/−^ fish showed fluorescence in the inner plexiform layer (IPL) and photoreceptor cell layer (PRL). The optic nerve (ON), optic chiasm (OC), and arborization fields of the retinotectal tract (AF) were immunoreactive. Scale bar 100 µm. **(D, E)** The immunoreactivity in the IPL and PRL of wild type TL (top) was reduced in *gjd2b* ^−/−^ */*Cx35.1^−/−^ larvae (bottom). Arrows indicate the IPL. Scale bars 50 µm.

The Tupfel longfin (TL) strain were used to generate the *gjd2b* ^*−/−*^/Cx35.1^−/−^ line in-house using the CRISPR-Cas9 system (Jinek et al., 2012; Li et al., 2016). Potential CRISPR target sites of the *gjd2b/*Cx35.1 gene were identified using the E-CRISP tool (www.e-crisp.org). A CRISPR sgRNA (Synthego, Redwood City, CA, United States of America) targeting a XhoI restriction site in exon one of the *gjd2b* gene was selected (Figure 1A). The sequence was: 5′-CUC​UUA​ACA​GGU​AAG​GGG​GU-3.’ CRISPR sgRNA/Cas9 complexes were formed at a 1:2 ratio supplemented with 0.05% phenol red prior to microinjections. For microinjection one-stage TL embryos were placed in a chilled chamber (2% agarose supplemented with E2 embryo medium) and subsequently injected with 2 nL of the Cas9:sgRNA duplex (400 pg). At 3dpf, injected larvae were genotyped. The remaining embryos of the founding generation (F0) were raised to adulthood. Founders were outcrossed to TL fish for two subsequent generations to reduce the risk of CRISPR off-target effects. Germline transmission was tested in each generation (F1-F2) and heterozygous progeny were raised to adulthood and in-crossed to generate the homozygous *gjd2b* ^*−/−*^/Cx35.1^−/−^ zebrafish line.

### 2.3 DNA extraction, PCR amplification, and genotyping

Zebrafish larvae (3dpf) or adult caudal fins were used for genotyping purposes. Adult zebrafish were anesthetized in pH-buffered 0.2 mg/mL tricaine methane sulfonate (MS-222) (Sigma-Aldrich, St. Louis, MO, United States of America) prior to caudal amputations with surgical scissors. Total DNA was extracted by incubation in 100 mM NaOH for 15 min at 95°C using with vigorous shaking. DNA samples were diluted in a 1:2 ratio with TE buffer (pH 8.0) prior to polymerase chain reaction (PCR) analysis. The *gjd2b* exon one region was amplified in a Mastercycler Nexus X1 (Eppendorf, Mississauga, ON, CA) using the primer information as follows: forward primer: 5′-GGT​TCT​CTG​TGT​TAC​ATT​CGC​CTC​C-3′ and reverse primer: 5′-CAA​TCA​TAG​TAG​AGT​GCT​GTT​GGA​CAG​C-3’. PCR primer pairs were ordered from IDT (Coralville, IA, United States of America) and optimized at an annealing temperature of 58°C. The PCR reaction was carried out using the Q5 High Fidelity DNA Polymerase PCR Kit (New England BioLabs, Ipswich, MA, United States of America) in a final volume of 20 µL consisting of 1 µL DNA-template and 19 µL Taq master mix. PCR thermal cycling conditions were initiated at 94°C for 15 min, followed by 38 cycles of denaturing at 94°C for 15 s, annealing at 58°C for 15 s, and extension at 72°C for 30 s. The final extension was carried out at 72°C for 10 min. To determine the presence of indel mutations, PCR amplicons were digested with XhoI using the Fast Digestion Top Fermentas Kit (Thermo Fisher Scientific; Burlington, ON, Canada) as per the manufacturer’s protocol. Amplicons were subsequently visualized on a 2% agarose gel supplemented with 0.005% ethidium bromide using the Alpha Imager HP System (Thermo Fisher Scientific; Burlington, ON, Canada). For Sanger sequencing, PCR amplicons were cloned into pJET1.2 using the CloneJET™ PCR Cloning Kit (Thermo Fisher Scientific; Burlington, ON, Canada) as per the manufacturer’s protocols. Constructs were transformed into DH5α competent *E. coli* cells. Plasmids were purified using the QIACube in combination with the QIAPrep spin miniprep kit (Qiagen, Germantown, MD, United States of America). Sequencing was performed by Eurofins Genomics (Toronto, Canada).

### 2.4 Quantitative real-time PCR (qRT-PCR) analysis

Total RNA (1 µg) was extracted and purified from pools of 30 zebrafish larvae at six to seven dpf using the RNeasy Plus Mini Kit (Qiagen, Germantown, MD, United States of America) as per the manufacturer’s protocols. When appropriate, larvae were collected immediately following exposure to behavioral tasks, and the tissue was frozen at −80°C until further analysis. Fish were homogenized by bead beating in 1xTE buffer (pH 8.0). RNAs were reverse transcribed into cDNA using the iScript Reverse Transcription Supermix (Bio-Rad, Mississauga, ON, Canada) as per the manufacturer’s instructions. Gene expression was analyzed by qRT-PCR using the SsoAdvanced universal SYBR Green Supermix (Bio-Rad, Mississauga, ON, Canada) in the CFX96™ Real-Time PCR Detection System (Bio-Rad, Mississauga, ON, Canada). Thermal cycling was carried out for 42 cycles of the following: 94°C for 30s, 52°C for 30s, and 72°C for 1min. Three housekeeping genes (*18S*, *tuba1a*, *actb2,* Supplementary Table S7) were used to determine the quality of the samples and for normalization purposes. CT values were averaged and exported from the CFX Manager Software (Bio-Rad, Mississauga, ON, Canada). The fold-difference for the relative gene expression was calculated in the Relative Expression Software Tool VS. 2009 (Pfaffl et al., 2002). Three technical replicates per gene were performed from three biological replicates. Primers are listed in Supplementary Tables S1, S3–5.

### 2.5 Immunohistochemistry

Six dpf larvae (TL, *gjd2b* ^*−/−*^/Cx35.1^−/−^) were humanely euthanized using MS222. The larvae were fixed in 4% paraformaldehyde (PFA) in 1xPBS overnight at 4°C, followed by cryoprotection in 30% sucrose in 1xPBS. After embedding in Tissue-Tek O.C.T, 10–15 µm sections were cut on a cryotome (Thermofisher Scientific, Burlington, ON, Canada). Sections were washed three times for 5 min with 1xPBS containing 0.1% Tween-20 (PBST) at RT. Unspecific binding sites were blocked with freshly prepared 5% normal goat serum (NGS, Sigma-Aldrich) in PBS-T for 1h at 4. Following blocking, samples were incubated with the primary mouse anti-Connexin 36 antibody (1:100, catalog #36-4600, Thermofisher, Burlington, ON, Canada) overnight at 4°C. Subsequent washes with PBS-T were for 1 hour at 4°C. Alexa 488 goat mouse secondary antibodies (1:3000 in 1% NGS PBS-T, Life Technologies, Burlington, ON, Canada) were applied for 1 h at RT. After three washes with PBS-T followed by one wash with PBS, specimens were mounted on microscope slides using ProLong Antifade with DAPI (Thermofisher, Burlington, ON, Canada). Confocal images were collected using LSM-ZEN2 software using a Zeiss LSM700 system (Carl Zeiss MicroImaging, Oberkochen, Germany) with a Plan-Apochromat 20x/NA0.8 or Plan-Apochromat 63x/NA1.3 oil DIC M27 objectives. The software optimized pixel resolution, line averaging, gain, and digital offset. The same imaging parameters were used to compare TL and *gjd2b* ^*−/−*^/Cx35.1^−/−^ tissues. The composite Figure 1 was created using Adobe Photoshop 2021.

### 2.6 Optical coherence tomography assay

A custom-developed spectral-domain optical coherence tomography (SD-OCT) system was built in Michelson configuration (Supplementary Figure S1), employing a superluminescent laser diode centered at 1,310 nm ( ± 75 nm at 10 dB; Exalos, Switzerland) and a 2048-pixel line scan camera spectrometer with a maximum acquisition rate of 147 kHz (Wasatch Photonics; United States of America). A 50/50 fiber coupler splits the source light into the reference and sample arms. In the sample arm, the output light illuminates the sample surface after passing through a reflective beam collimator (Thorlabs; United States of America), a 2-DOF galvo mirror, and an objective lens (LSM02, Thorlabs; United States of America). The 2-DOF Galvo mirror allows for collection of reflected light from the sample while raster scanning sample surface. In the reference arm, a polarization controller, a dispersion compensation block, and a gold-coated reference mirror were installed. The back-reflected light of these two arms is subsequently merged after passing through the beam splitter and redirected to the spectrometer by the optical circulator. The formed interference pattern in the spectrometer is captured by a line scan camera. The captured signal is digitized and sent to the computer for processing. To form an A-line (i.e., depth profile of sample reflections at a given point on sample surface), the tomograms of the sample, the spectrometer signal is background subtracted and mapped to k-space before applying Fourier transformation to calculate depth profile of reflectors in the z-space (physical depth space). Aforementioned processes are repeated for data acquired during raster scanning to eventually form 3-D OCT tomograms of zebrafish eye. The axial and lateral resolutions of the system in tissue were measured as 8.5 µm and 10 μm, respectively.

Before imaging age-matched adult zebrafish were humanely euthanized using MS222 and fixed in 4% paraformaldehyde (PFA) in 1xPBS overnight at 4°C. Fixed zebrafish (n = 26/genotype) were placed in a silicon mold to orient the eye toward OCT system’s objective lens. To minimize specular reflections from sample surface, a thin layer of PBS (∼80 µm) was placed over the eye before imaging. All captured OCT tomograms were interrogated in ImageJ software which enabled measurement of various geometrical parameters (see Supplementary Table S3) through a standard calibration process.

### 2.7 Zebrafish body length and head measurements

Zebrafish embryos were raised in 10 cm ϕ Petri-dishes in population-matched groups. At 7dpf, individual larvae were imaged by light microscopy (IX81; Olympus, Toronto, ON, Canada). To minimize human experimental error, three measurements for body length, head length, and midbrain diameter were acquired for each image using ImageJ. Measurements for each image were subsequently averaged before reporting.

### 2.8 Behavioral assays

At 6dpf, larvae were acclimated to the behavioral room for 24 h. On the morning of testing (7dpf), individual larvae were transferred into 48- or 96-well plates (Greiner) (for free-swim, VMR, and FSTR) filled with egg water. Transparent plates were utilized for all experiments. The cross-maze to assess color vision was generated in-house. Behavioral assays were performed using the Zebrabox^®^ system and the Zebralab analytical software (Viewpoint Life Technology, Lyon, Fr) to obtain automated tracking data from video files. The recording chamber holding the plates was equipped with a water heating and flow system to maintain the temperature at 28°C. Illumination was from the bottom with LED and infrared lights. Unless otherwise stated, larvae underwent a dark-adaptation period for 2 hours prior to behavioral analyses. All experiments utilizing light were performed at 30% light intensity unless otherwise stated, where the Zebrabox^®^ system was capable of 8,000 lux at 100%. Data was acquisitioned in 1s, 10s, 1 min, or 5min intervals for time periods ranging from 5 min to 4.5 h as outlined in figures. Data was acquisitioned in 1s, 10s, 1 min, or 5min intervals for time periods ranging from 5 min to 4.5 h as outlined in figures. The experimental designs for VMR (Emran et al., 2008), Color vision (Park et al., 2016), were adapted from previous reports. Activity was categorized by inactivity (0.0 mm/s), coast swimming (between 0.0 mm/s and 20.0 mm/s) and burst swimming (>20 mm/s). Where applicable, results were normalized in a three step process to account for differences in larval activity caused by 1) variation in light-intensity received by each well, 2) the batch effect from the inclusion of experimental replicates and 3) variation in the baseline activity measured during the final 5 min of light or dark adaptation based on a previous report (Xie et al., 2019).

#### 2.8.1 Spontaneous/free swim assay

Spontaneous zebrafish larval locomotor activity was measured under constant illumination (30%) or darkness (0%). For constant illumination, larvae were acclimated in light (30%) for 2 h prior to behavioral analysis. Locomotor activity was tracked for 5 min or 1 h, depending on the type of data acquisition. Data were normalized accordingly.

#### 2.8.2 Visual Motor Response (VMR) assay

Responsiveness to light changes was measured and under mesopic conditions as described (Emran et al., 2008). Larvae were dark-adapted for 2 h prior to the experiment commencing. Locomotor activity was tracked for 3.5 h (3 h of the experimental protocol and 30 min prior to illumination) for the standard set-up. For mesopic VMR, locomotor activity was tracked for 2.5 h (2 h of the experimental protocol and 30 min prior to illuminations). For both assays, two to three trials of alternating lighting conditions (30 min Light-ON; 30 MIN light-OFF) were utilized. Light-ON intensity for mesopic VMR was set at 5% as indicated in the results text. Mean activity, quantified by the burst duration s), was measured 1s following light transition. Data were normalized accordingly.

#### 2.8.3 Innate color preference test

The innate color preference test allowed for color bias and color-blindness to be assessed in zebrafish larvae. Here, a custom-made cross maze was milled from 5 mm transparent acrylic sheets. Each arm of the maze was of equal dimensions (15 × 35 × 10 mm, W × L ×H) and could accommodate removable sleeves of different colors (B-blue, G-green, Y-yellow, R-red, Null-no color). The test was performed in two variations. In the first set larvae were exposed to all four colors simultaneously. In the second test larvae were exposed to two colors and a neutral stimulus (white light). Up to 20 larvae of the same genotype were placed into the center of the cross maze. Larvae were dark-adapted to avoid pre-exposure to the colors, and color sleeves were rotated to avoid location bias. Locomotor activity was tracked for 40 min (35 min of illumination and 5 min prior to illumination). Data was measured manually from tracking videos by counting the number of fish in each arm every 2 min for the duration of the experiment.

#### 2.8.4 Flash stimuli threshold response (FSTR) assay

Responsiveness to light flashes was assessed with the FSTR assay. Various stimulus lengths were sequentially tested (in ms, 10, 20, 50, 100, 250, 500, 1,000]); however, the final analysis utilized the 10 m and 1000 m experimental endpoints. The experimental design began with 2 h dark adaptation and was followed by alternating a light cue with a 20-min rest period in darkness To avoid habituation to the stimuli, cue lengths were presented in forward and reverse order. Larval locomotor activity was tracked for 2.5hrs; the last 5 min of adaptation period prior to the presentation of the initial light cue was used in normalization calculations. Mean responsiveness, quantified as burst duration s), 1 s following each stimulus was measured. Data were normalized accordingly.

#### 2.8.5 Statistical analysis

Unless otherwise stated, all data is presented as the mean ± SEM. The sample size for behavioral studies varied from n = 48 to n = 144 across a minimum of two experimental replicates; sample sizes are indicated in the figures. Statistical significance was determined by using a two-tailed unpaired Student’s t-test or its non-parametric equivalent Mann-Whitney, one-way ANOVA, or its non-parametric equivalent or Kruskal–Wallis, or two-way ANOVA with a Tukey *post hoc* test where appropriate. Normality was tested with the Shapiro-Wilk normality test. Statistical significance was defined by a *p*-value of ≤0.05. Statistical analyses were performed in GraphPad Prism 6 (GraphPad Software, La Jolla, CA, United States of America). Gardner-Altman estimation plots were created in a R-statistical program (The R Foundation, Aukland, New Zealand; https://cran.r-project.org/web/packages/dabestr/vignettes/using-dabestr.html); they show the mean difference between groups. Both groups are plotted on the left or top axes; the mean difference is plotted on floating axes on the right or bottom as a bootstrap sampling distribution. The mean difference is depicted as a dot; the 95% confidence interval is indicated by the ends of the vertical error bar. All remaining figures were created in GraphPad Prism 6.

## 3 Results

### 3.1 Generation and primary characterization of the *gjd2b* ^*−/−*^ */*Cx35.1^−/−^ line

The Cx35.1 knock-out (*gjd2b* ^−/−^ **/**Cx35.1^−/−^) model was created utilizing a CRISPR-Cas9 genome engineering strategy and screening for indel mutations generated by the non-homologous end joining (NHEJ) repair system. Exon one of the *gjd2b* gene was targeted with a sgRNA located upstream of the start codon (Figure 1A). A founder (F0) with a minimal 1bp substitution (position G12 →A) was chosen because this mutation altered amino acid 4 (tryptophan 4) to an early stop codon (Figures 1A,B). The F0 mutation was germline transmitted and after outcrossing with TL for two generations the homozygous *gjd2b* ^−/−^/Cx35.1^−/−^ line was established in the F3 generation.

Previous reports have demonstrated the expression of Cx35 in the zebrafish and perch retina (McLachlan et al., 2003; O'Brien et al., 1998); however, these results were likely confounded by the co-expression of closely related Cx35 proteins. With no access to antibodies discriminating the four zebrafish Cx35 orthologs *gjd1a/*Cx34.1, *gjd2b/*Cx35.1, *gjd1b/*Cx34.7, and *gjd2a/*Cx35.5, we investigated the Cx35.1^−/−^ phenotype by visualizing changes in the immunofluorescence localization patterns detectable with a monoclonal anti-Cx36 antibody in 6-day post fertilization (6dpf) larvae (Figure 1C). The antibody detected Cx35/36 immunoreactivity in the retina, the optic nerve, and the arborization fields where axons from retinal ganglion cells are expected to contact dendrites from neuronal cells in the retinotectal pathway. A reduction in the fluorescence signal was observed in the inner plexiform layer (IPL) and between photoreceptor cells (PRCs) of *gjd2b* ^−/−^ */*Cx35.1^−/−^ larvae. The residual signals in the remaining PRCs and throughout the retina was attributed to the expression of other Cx35 orthologs that we could not positively identify by immunohistochemistry.

Following the confirmation of the Cx35.1^−/−^ genotype and partial loss of Cx35/36 immunoreactivity, we tested whether targeted ablation of the *gjd2b* gene causes a compensatory regulation of connexins in larvae. A previous RNA-seq analysis of 6dpf WT larvae had demonstrated the expression of two of the four zebrafish Cx36 orthologs; *gjd1a/*Cx34.1 and *gjd2b/*Cx35.1 were detected, but not *gjd1b/*Cx34.7 or *gjd2a/*Cx35.5 (GEO database entry GSE181853; (Whyte-Fagundes et al., 2022). Having a key interest in the role of Cx35.1 in vision, the expression of all four Cx36 orthologs and nine other connexin genes expressed in the eye were quantified (Supplementary Table S1) (Cheng et al., 2003; Zoidl et al., 2008; Yoshikawa et al., 2017). The RNA transcript levels of this representative subset of connexins expressed in the retina was compared between WT and Cx35.1^−/−^ larvae using qRT-PCR (Supplementary Figure S2). In comparison to WT larvae, the expression of *gjd1b/*Cx34.7 was reduced (0.680-fold, *p* = 0.014). Both *gjd1b/*Cx34.7 and *gjd2b/*Cx35.1 are co-expressed in cone photoreceptors (O'Brien et al., 1998; O'Brien et al., 2004), forming heterotypic gap junctions (Rash et al., 2015) which form the molecular basis of functional asymmetry of electrical synapses (Rash et al., 2013). The loss was evidence for altered visual functions. Furthermore, a reduction of *gja2*/Cx39.9 (0.314-fold, *p* = 0.005) and *gja3*/Cx48.5 (0.608-fold, *p* = 0.022) expression was detected. The expression of other connexins were indistinguishable from the WT. The regulation of *gja2*/Cx39.9 (Cx46 in human and murine models), and *gja3*/Cx48.5 (Cx50 in human and murine models) was of interest due to the role of both connexins in ocular lens homeostasis (Cheng et al., 2003; Brink et al., 2020). In summary, the initial characterization of *gjd2b* ^−/−^/Cx35.1^−/−^ larvae showed evidence that the depletion of Cx35.1 protein expression affected the expression of other connexins with roles in the communication between PRCs and in the zebrafish lens.

### 3.2 *Gjd2b/*Cx35.1 depletion altered ocular dimensions, inducing hyperopic shifts in adult zebrafish

**FIGURE 2:**
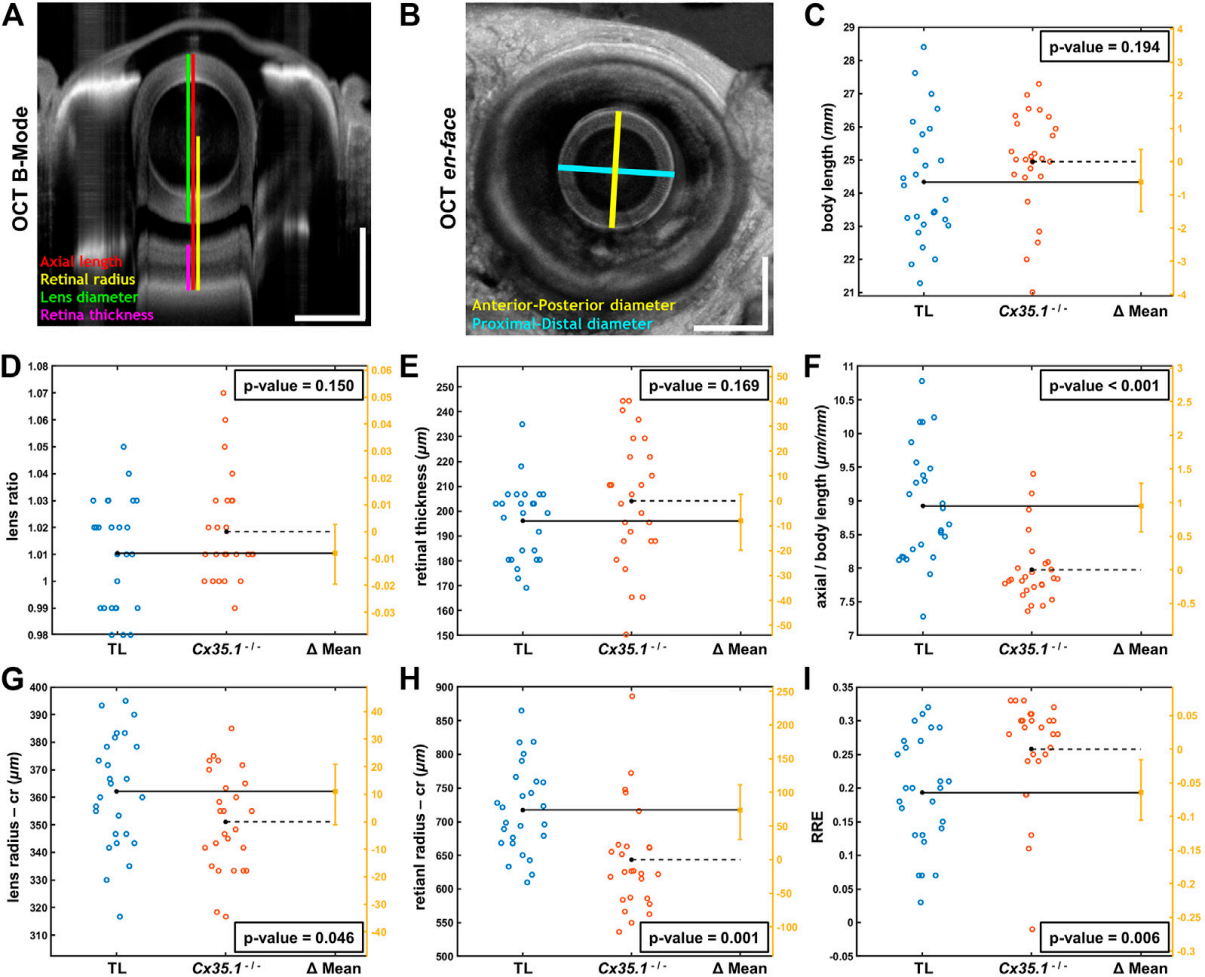
Shortening of the Eye Axial Length Induces a Hyperopic Shift in Adult *Gjd2b*/Cx35.1^−/−^ Fish. Representative SD-OCT **(A)** B-mode and **(B)** *en face* images of a zebrafish eye (scale bars = 500 µm); geometrical parameters extracted from the OCT datasets are shown and annotated with different colors; the spherical lens is seen as an oval in the B-mode image (panel A) due to a significantly higher refractive index of lens compared to soft tissue. The quantification of **(C)** the body length (mm), **(D)** the lens ratio (µm/µm) as a measure of circulatiry, and **(E)** the retinal thickness (µm) revealed no statistically significant difference between TL controls and *gjd2b* ^*−/−*^ */*Cx35.1^−/−^. However, the **(F)** axial length (from top of the lens to the RPE) normalized by body length (µm/mm), the **(G)** lens radius (µm), and the **(H)** retinal radius (from the center of the lens to the RPE; µm) collectively showed significantly smaller values (i.e., reduction in size of the eye) in *gjd2b* ^*−/−*^/Cx35.1^−/−^. The **(I)** calculated relative refractive error (RRE; see text for definition) was significantly higher in *gjd2b* ^*−/−*^/Cx35.1^−/−^ which suggested a hyperopic shift, presumably, due to loss of *gjd2b*/Cx35.1 function. The sample size (N) was 26 for each of the TL and *gjd2b* ^*−/−*^ */*Cx35.1^−/−^ adult fish groups.

In light of a recent report linking *gjd2b/*Cx35.1 to the development of refractive errors and nuclear cataracts (Quint et al., 2021), the eyes of age-matched adult zebrafish were imaged with spectral domain optical coherence tomography (SD-OCT) (Supplementary Figure S3). Using OCT B-mode and *en face* images (Figures 2A,B), six primary parameters were quantified (Supplementary Table S2). To prevent biases, these geometrical parameters were quantified either as ratios or as values normalized by the individual measured body lengths of WT (strain: TL) controls and *gjd2b* ^−/−^/Cx35.1^−/−^ (in mm, TL: 24.33 ± 1.84; Cx35.1^−/−^: 24.94 ± 1.52; *p* = 0.194, n = 26/genotype). The weight of TL controls and *gjd2b* ^−/−^/Cx35.1^−/−^ (in Gram, TL: 0.27 ± 0.08; Cx35.1^−/−^: 0.29 ± 0.05; *p* = 0.358, n = 26/genotype) were also measured; these measurements, however, were not used for normalization of measured metrics as they were deemed unreliable due to the inherent variations caused by the fixation process.

Statistical analysis of quantified parameters in Figure 2 C-I revealed no significant difference between ratio of proximal-distal and anterior-posterior lens diameters (i.e., lens ratio) of TL controls and *gjd2b* ^−/−^/Cx35.1^−/−^ (in micrometer/micrometer, TL:1.01 ± 0.02; Cx35.1^−/−^: 1.02 ± 0.02; *p* = 0.150, n = 26/genotype); similar trend was observed in the measured retinal thicknesses of TL controls and *gjd2b* ^−/−^/Cx35.1^−/−^ (in micrometer, TL: 196.07 ± 15.17; Cx35.1^−/−^: 204.16 ± 25.36; *p* = 0.169, n = 26/genotype). No evidence for the occurrence of cataracts were found in any of the TL or *gjd2b* ^−/−^/Cx35.1^−/−^ fish. The comparative analysis, however, demonstrated that mutants and TL differed significantly in terms of normalized eye axial length (in micrometer/millimeter, TL: 8.92 ± 0.86; Cx35.1^−/−^: 7.97 ± 0.50; *p* < 0.001, n = 26/genotype), lens radius (in micrometer, TL: 362.12 ± 20.50; Cx35.1^−/−^: 351.15 ± 17.99; *p* = 0.046, n = 26/genotype), and retinal radius (in micrometer, TL: 717.52 ± 66.23; Cx35.1^−/−^: 643.52 ± 78.40; *p* = 0.001, n = 26/genotype); see Supplementary Table S2.

The statistically different geometrical parameters, collectively, point to reduction in size of the eye of compared to those of the TL fish which may result in hyperopic shifts. To evaluate this hypothesis, similar to other relevant OCT studies (Collery et al., 2014), Relative Refractive Error (RRE) was calculated for TL controls and mutants as 1- (retinal radius/F); here, parameter F is an idealized focal length equal to the measured lens radius×2.324. The significance of RRE values is that they offer a normalized/unitless metric with values lower than zero for myopic eyes (i.e., when distance from lens center to RPE is greater than the expected retinal radius) and values greater than zero for hyperopic eyes (i.e., when distance from lens center to RPE is less than the expected retinal radius). Accordingly, an increase in RRE value highlights a hyperopic shift, while a decrease in RRE value suggests myopic shift (Collery et al., 2014). Statistical comparison of calculated RREs demonstrated a statistically meaningful increase in RRE values of *gjd2b* ^−/−^/Cx35.1^−/−^ over the TL controls (in micrometer/micrometer, TL: 0.19 ± 0.08; Cx35.1^−/−^: 0.26 ± 0.08; *p* = 0.006, n = 26/genotype); the positive values of RRE for the TL controls were due to the fixation process which makes the eye slightly hyperopic as reported by others (Collery et al., 2014). The above analysis confirmed our hypothesis that loss of *gjd2b/*Cx35.1 function contributed to a hyperopic phenotype in adult fish.

### 3.3 *Gjd2b*/Cx35.1 regulates wnt and dopamine receptor gene expression

**FIGURE 3:**
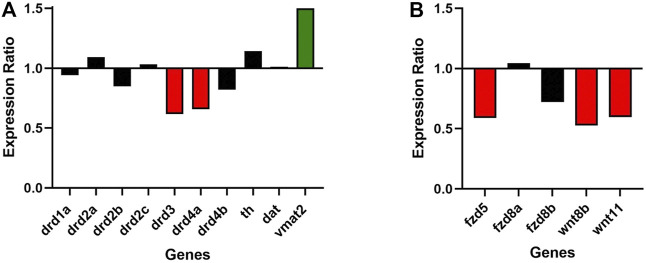
*Gjd2b*/Cx35.1 regulates Wnt and dopamine receptor gene expression. A qRT-PCR analysis of selected zebrafish dopamine **(A)** pathway genes showed the differential regulation of the D2 family receptors *drd3* and *drd4a*, and *vmat2* (N = 3). **(B)** Shows the downregulation of Wnt/frizzled pathway genes *fdz5*, *wnt8b*, and *wnt11* (N = 4).

Roles of dopaminergic signaling and the Wnt/ß-catenin signaling in neurodevelopment have been demonstrated (*Panula et al., 2010*; *Sutton et al., 2021*). Since Cx36 regulates neuronal differentiation from neural progenitor cells (*Hartfield et al., 2011*) we tested whether dopamine receptor gene expression was modulated in 6dpf gjd2b^−/−^/Cx35.1^−/−^ larvae. The expression of zebrafish dopamine receptor (*drd*) subtypes (*drd1, drd2a/b/c, drd3, drd4a/b*) as well as the dopamine transporter (*dat*) were quantified by qRT-PCR. The tyrosine hydroxylase (*th*) and the vesicular monoamine transporter (*vmat2*) genes were included in the analysis because of the roles in presynaptic dopamine production and in the regulation of packaging and subsequent release of dopamine vesicles into the synaptic cleft (Supplementary Table S3). In comparison to the WT, we found a significant downregulation of *drd3* (0.616-fold, *p* = 0.007) and *drd4a* receptor genes (0.657-fold, *p* < 0.001), and an upregulation of *vmat2* (1.501-fold, *p* = 0.003) (Figure 3A; Table 1)**.** Expression levels of the other transcripts were indistinguishable from the WT. The regulation of presynaptic *vmat2* and postsynaptic *drd3* and *drd4a* receptors suggested a concerted transsynaptic alteration of synaptic communication. Considering this, we concluded that the regulation of dopaminergic genes reflected a mechanism in which *vmat2* upregulation compensates functional deficits of dopaminergic synapses utilizing D2-receptor family genes.

**TABLE 1:** qRT-PCR results for dopamine-related genes in TL and *gjd2b* ^*−/−*^/Cx35.1^−/−^ larvae (N = 3 replicates).

Gene	RT-qPCR expression (rest)	S.E. (rest)	*p*-value (rest)	Result
*18s*	1.190			
*actb1*	0.878			
*tuba1b*	0.957			
*drd1a*	0.940	0.768–1.174	0.361	
*drd2a*	1.093	0.905–1.303	0.118	
*drd2b*	0.849	0.645–1.241	0.133	
*drd2c*	1.031	0.761–1.359	0.754	
*drd3*	0.616	0.364–0.895	0.007	DOWN
*drd4a*	0.657	0.397–0.909	0.000	DOWN
*drd4b*	0.820	0.479–1.511	0.255	
*th*	1.142	0.599–1.911	0.430	
*dat*	0.996	0.746–1.440	0.970	
*vmat2*	1.501	0.980–2.312	0.003	UP

Experimental evidence and genome-wide association studies imply the canonical Wnt signaling pathway in eye development and eye disorders such as myopia (Cheng et al., 2013; Liu et al., 2021). A potential correlation between Cx35.1 and the canonical Wnt-signaling pathway affecting eye-development was testable based on a previous RNA-seq expression analysis showing expression of wingless-type MMTV integration site family and frizzled class receptor genes in 6dpf TL larvae (GEO database entry GSE181853) (Supplementary Table S4). A RT-qPCR analysis confirmed the downregulation of *wnt8b* (0.527-fold, *p* = 0.024), *wnt11* (0.596-fold, *p* = 0.013), and *fdz5* (0.690-fold, *p* = 0.014) in *gjd2b* ^*−/−*^ */*Cx35.1^−/−^ larvae (Figure 3B; Table 2). The three genes are known for roles in camera eye development. The genes *fdz8a* and *fdz8b* were not significantly regulated. We concluded that deregulation of the canonical Wnt signaling pathway could be a contributing factor to hyperopia in adult *gjd2b* ^*−/−*^ */*Cx35.1^−/−^ fish. The results provide evidence for a crosstalk between dopamine D_2_ type-receptors, the Wnt/β-catenin signaling pathways, and Cx35.1.

**TABLE 2:** qRT-PCR results for Wnt/frizzled pathway genes in TL and *gjd2b* ^*−/−*^/Cx35.1^−/−^ larvae (N = 4 replicates).

Gene	RT-qPCR expression (rest)	S.E. (rest)	*p*-value (rest)	Result
18s	*1*			
fdz5	*0.590*	0.285–1.111	0.014	DOWN
fdz8a	1.044	0.569–1.845	0.783	
fdz8b	0.722	0.389–1.365	0.079	
wnt8b	0.527	0.237–1.167	0.024	DOWN
wnt11	0.596	0.318–1.129	0.013	DOWN

### 3.4 Loss of *gjd2b/*Cx35.1 caused a dysmorphic head feature

**FIGURE 4:**
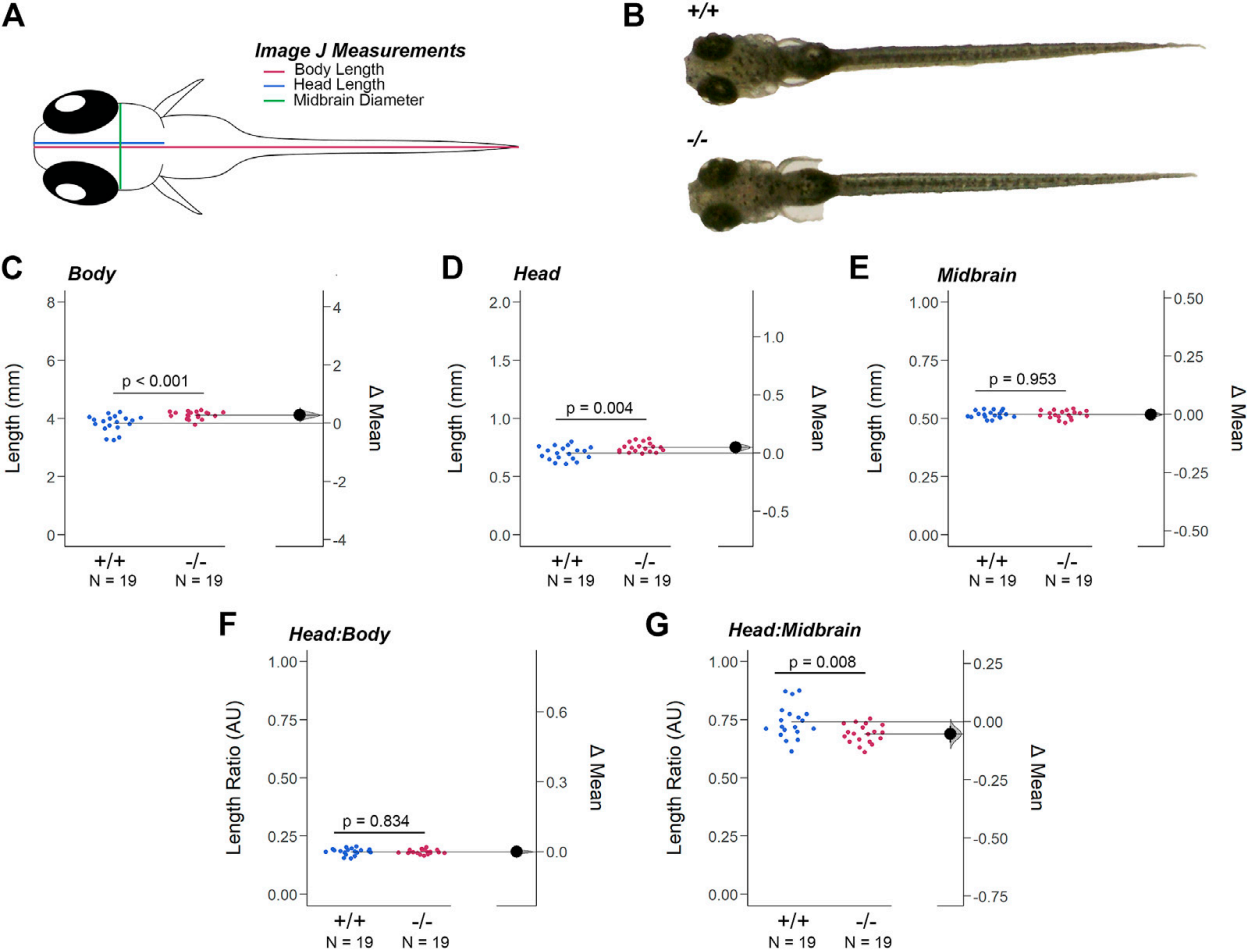
Loss of *Gjd2b*/Cx35.1 Caused a Dysmorphic Head Feature. **(A)** Illustration of the zebrafish measurements taken in Image J. **(B)** Images of 7dpf wild-type TL and *gjd2b*/Cx35.1^−/−^ zebrafish larva. Quantification of the **(C)** body length, **(D)** head length, and **(E)** midbrain diameter. *Gjd2b* ^*−/−*^ */*Cx35.1^−/−^ larvae were larger in both body and head length in comparison to the age-matched wild-type larvae. The **(F)** Head-to-Body ratio and **(G)** Head-to-Midbrain ratios demonstrated a reduction in the midbrain diameter in *gjd2b* ^*−/−*^ */*Cx35.1^−/−^ larvae relative to their body size, suggesting a developmental alteration. The statistical significance was determined by the Mann-Whitney test, *p* ≤ 0.05. **(C–G)** Shows Estimation Plots. The sample size (N) was 19 for WT and *gjd2b* ^*−/−*^ */*Cx35.1^−/−^ larvae.

The resolution of the OCT equipment prevented the analysis of the larval retina, but at 7dpf additional morphological alterations to head-to-tail (body) length, head length, and midbrain diameter (MD) were detected (Figures 4A,B). The body and head length of *gjd2b* ^*−/−*^ */*Cx35.1^−/−^ larvae was increased when compared to the WT (in mm, *Body Length* - WT: 3.84 ± 0.06, n = 19, Cx35.1^−/−^: 4.12 ± 0.03, n = 19, *p* = 0.0003; *Head Length* - WT: 0.70 ± 0.01, Cx35.1^−/−^: 0.75 ± 0.01, *p* = 0.0038, *Mann-Whitney* test). The MD was indistinguishable between the two genotypes (in mm, WT: 0.52 ± 0.003, Cx35.1^−/−^: 0.52 ± 0.004, *p* = 0.95) (Figures 4C–E).

These findings led us to investigate whether the differences in size were balanced by measuring the head-to-body and head-to-midbrain ratios. No significant differences in the head-to-body ratio were detected, suggesting that at 7dpf the *gjd2b/*Cx35.1^−/−^ larvae were objectively larger in their body and head length (WT: 0.18 ± 0.003, Cx35.1^−/−^: 0.18 ± 0.002, *p* = 0.84) (Figure 4 F). In contrast, the head-to-midbrain ratio of *gjd2b/*Cx35.1^−/−^ larvae was smaller (WT: 0.74 ± 0.71, Cx35.1^−/−^: 0.69 ± 0.01, *p* = 0.008) (Figure 4G). This suggested that *gjd2b/*Cx35.1 expression affected larval growth and development. The reduction of the midbrain diameter detected in *gjd2b* ^*−/−*^ */*Cx35.1^−/−^ larvae suggested functional consequences to visual sensory integration and motor output pathways in knock-out larvae.

### 3.5 Loss of *gjd2b*/Cx35.1 altered larval dominant swimming competence in darkness

**FIGURE 5:**
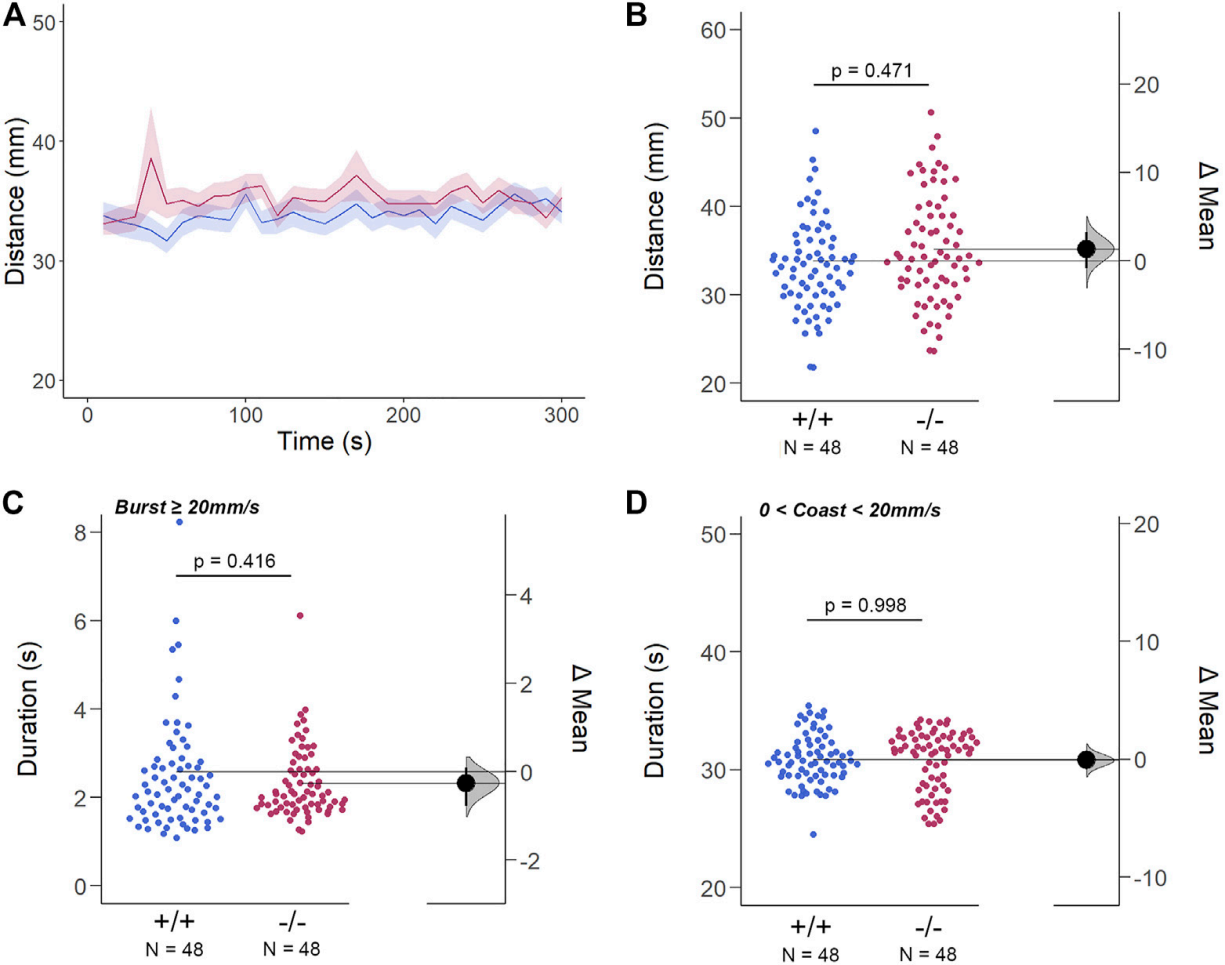
The Spontaneous Swimming Activity of Zebrafish Larvae in Continuous Light (Light-ON). **(A–D)** Spontaneous larval swimming activity was assessed in a free-swimming assay under light (light-ON) conditions for 5 min **(A, B)** The distance larvae swam (mm) lacking the *gjd2b*/Cx35.1 gene was indistinguishable from the WT. **(C, D)** The duration (s) of larval swimming bouts was assessed by analyzing the burst and coast swimming modalities separately. *Gjd2b* ^*−/−*^ */*Cx35.1^−/−^ larvae were indistinguishable from the WT. The statistical significance was determined by the Two-Way ANOVA test with the light-OFF dataset, *p* ≤ 0.05. The sample size (N) was 72 for WT-type and *gjd2b* ^*−/−*^ */*Cx35.1^−/−^ larvae.

**FIGURE 6:**
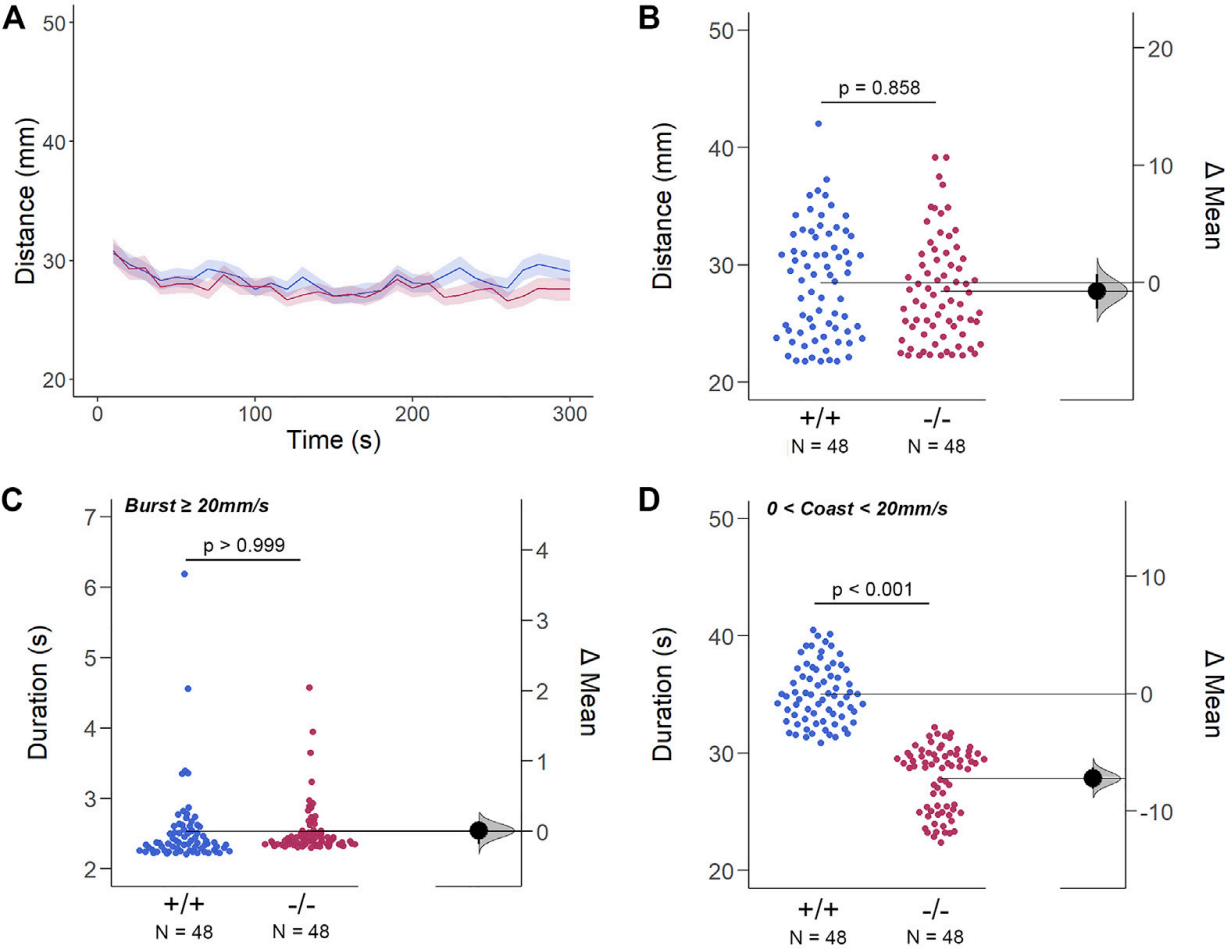
The Spontaneous Swimming Activity of Zebrafish Larvae in Continuous Dark (Light-OFF). **(A–D)** Spontaneous larval swimming activity was assessed in a free-swimming assay under dark (light-OFF) conditions for 5 min **(A, B)** The distance larvae swam (mm) lacking the *gjd2b*/Cx35.1 gene was indistinguishable from the WT. **(C, D)** The duration (s) of larval swimming bouts was assessed by analyzing the burst and coast swimming modalities separately. *Gjd2* ^*−/−*^ */*Cx35.1^−/−^ larvae demonstrated a significant reduction in the coast duration under darkness in comparison to the WT, suggesting that dominant swimming competence was compromised in a light-dependent manner. All else was indistinguishable from the WT. The statistical significance was determined by the Two-Way ANOVA test with the light-ON dataset, *p* ≤ 0.05. The sample size (N) was 72 for WT-type and *gjd2b* ^*−/−*^ */*Cx35.1^−/−^ larvae.

The structural and genetic changes detected in *gjd2b* ^*−/−*^ */*Cx35.1^−/−^ larvae suggested visual-motor deficiencies causing testable behavioral phenotypes. To address this question, spontaneous locomotor activity was quantified in 7dpf zebrafish in light-ON or light-OFF conditions. Both WT and *gjd2b* ^*−/−*^ */*Cx35.1^−/−^ larvae increased the swimming activity in light-ON conditions relative to light-OFF (WT: *p* < 0.001; Cx35.1^−/−^: *p* < 0.001; both n = 72). However, the swimming distance of *gjd2b* ^*−/−*^ */*Cx35.1^−/−^ larvae and WT larvae was indistinguishable in either light-ON (Figures 5A,B) and light-OFF conditions (Figures 6A,B) (mm/10s, light-OFF - WT: 28.49 ± 0.56, n = 72; Cx35.1^−/−^: 27.76 ± 0.52, n = 72, *p* = 0.858; light-ON - WT: 33.85 ± 0.78; Cx35.1^−/−^: 35.19 ± 0.71, *p* = 0.471, Two-Way ANOVA).

Next, differences in swim bouts were investigated. Larvae utilize a combination of slow rhythmic and fast burst bouts, known as the burst-glide swimming style, during free swim (*Muller et al., 2008*; *Voesenek et al., 2018*). Mechanisms underlying slow rhythmic (swim speeds less than 20 mm/s) and fast burst (speeds ≥20 mm/s) bouts represent distinct motor neuron circuits and subsequent muscle recruitment. Therefore, both dominant (coast) and subordinate (burst) swimming competence, mediated by slow motor neurons and Mauthner neurons, respectively, were quantified separately during free swim.

For subordinate swimming competence, both WT and *gjd2b* ^*−/−*^ */*Cx35.1^−/−^ larvae were indistinguishable in light-ON (in s, WT: 2.53 ± 0.07, Cx35.1^−/−^: 2.54 ± 0.04, *p* > 0.999) (Figure 5 C) and light-OFF conditions (in s, WT: 2.58 ± 0.20, Cx35.1^−/−^: 2.33 ± 0.09, *p* = 0.416) (Figure 6C). Similarly, in light-ON conditions, the coast duration of knock-out larvae were indistinguishable from the WT (in s, WT: 30.90 ± 0.25, Cx35.1^−/−^: 30.82 ± 0.30, *p* = 0.998) (Figure 5D). However, significant reduction in the coast duration in *gjd2b* ^*−/−*^ */*Cx35b^−/−^ larvae was detected in light-OFF conditions (in s, WT: 35.07 ± 0.30, Cx35.1^−/−^: 27.86 ± 0.32, *p* < 0.001) (Figure 6D). The results showed that the consequence of *gjd2b/*Cx35.1 depletion represented a change to the larval dominant swimming competence that was limited to darkness.

### 3.6 Cone photoreceptor cell activity is enhanced in *gjd2b* ^*−/−*^/Cx35.1^−/−^ larvae

**FIGURE 7:**
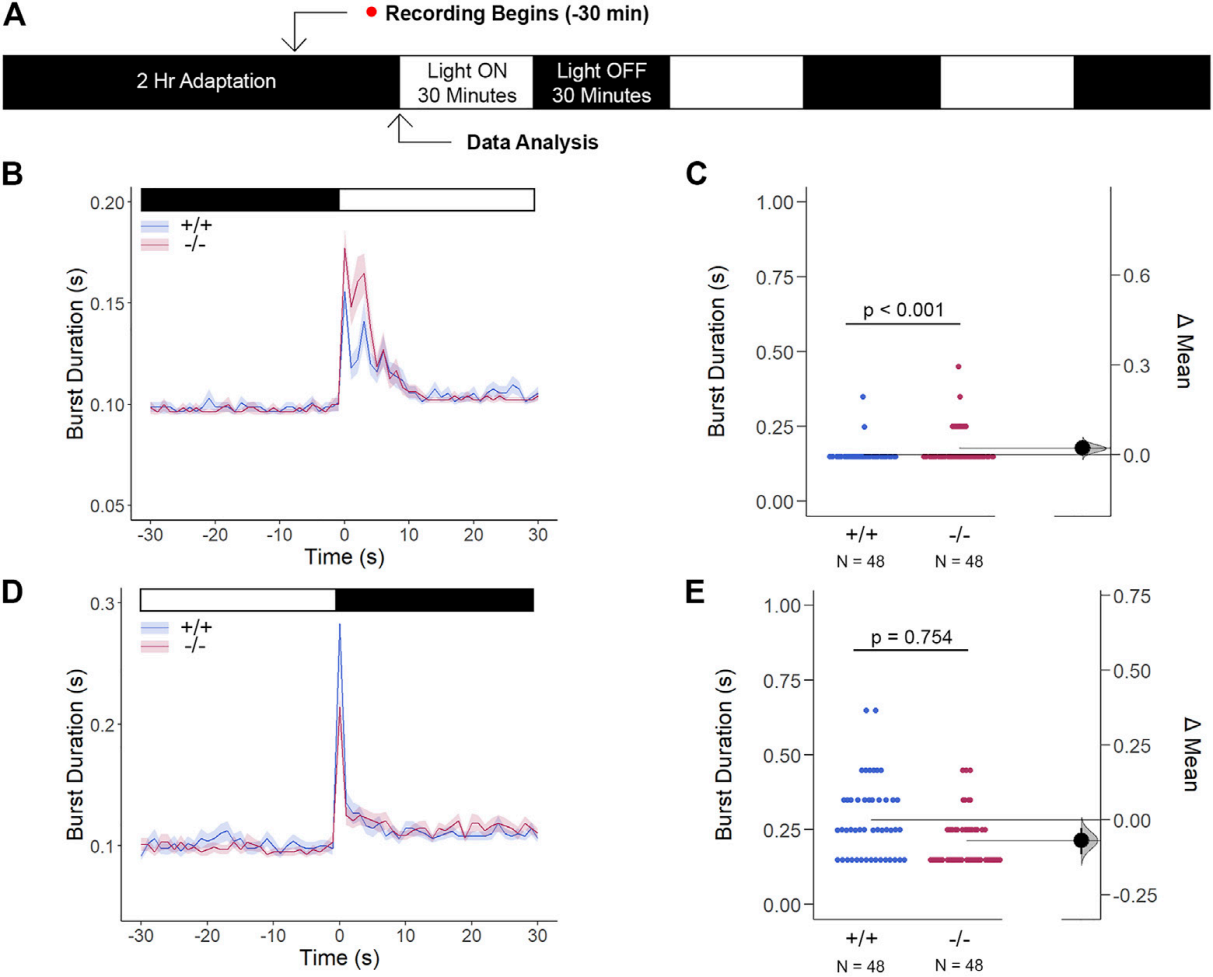
The Cone Photoreceptor Cell Activity is Enhanced in *gjd2b* ^*−/−*^/Cx35.1^−/−^ Larvae. **(A)** Overview of the experimental procedure. Burst activity was assessed for **(B, C)** Light-ON responses and **(D, E)** Light-OFF responses. Both WT and *gjd2b* ^*−/−*^ */Cx35.1* ^*−/−*^ larvae readily detected light transitions indicative of visual functionality. In comparison to the WT, *gjd2b* ^*−/−*^ */*Cx35.1^−/−^ larvae were hyperactive, suggesting that they were more sensitive to light stimulation. No significant difference was found for light-OFF responses. The statistical significance was determined by a Mann-Whitney test. The sample size (N) was 48 for wild-type and *gjd2b* ^*−/−*^ */*Cx35.1^−/−^ larvae.

Since we previously confirmed that the loss of *gjd2b* ^*−/−*^ */*Cx35.1^**−/−**^ altered retinal expression patterns, which was consistent with genetic profiles alluding to dysregulated eye development, we tested whether the retina was functionally intact using the standardized Visual Motor Response (VMR) assay (Figure 7A). Due to the nature of the startle/escape behavior teleost species exhibit in response to a sudden change in light intensity, we measured the burst activity as an indication of changes in retinal signaling. Both genotypes demonstrated an immediate and robust increase in burst duration in response to the light-ON VMR transitions. *Gjd2b* ^*−/−*^ */*Cx35.1^−/−^ larvae were hyperactive in comparison to the WT (in s, WT: 0.16 ± 0.01, Cx35.1^−/−^: 0.18 ± 0.01, n = 48, *p* < 0.001, Mann-Whitney) (Figures 7B,C). In the light-OFF condition, the VMRs were indistinguishable (in s, WT: 0.28 ± 0.02, Cx35.1^−/−^: 0.21 ± 0.01, *p* = 0.754) (Figures 7D,E). The results showed that *gjd2b* ^*−/−*^ */*Cx35.1^−/−^ larvae readily detected light changes and were most sensitive to photopic light stimuli. This suggested that retinal signaling routed to cone photoreceptors, and downstream acting pathways were altered in *gjd2b* ^*−/−*^ */*Cx35b^−/−^ larvae. We hypothesized, however, that signaling to rod photoreceptors would be preserved.

### 3.7 *Gjd2b* ^*−/−*^ */*Cx35.1^−/−^ larvae have normal threshold responses to flash stimuli

**FIGURE 8:**
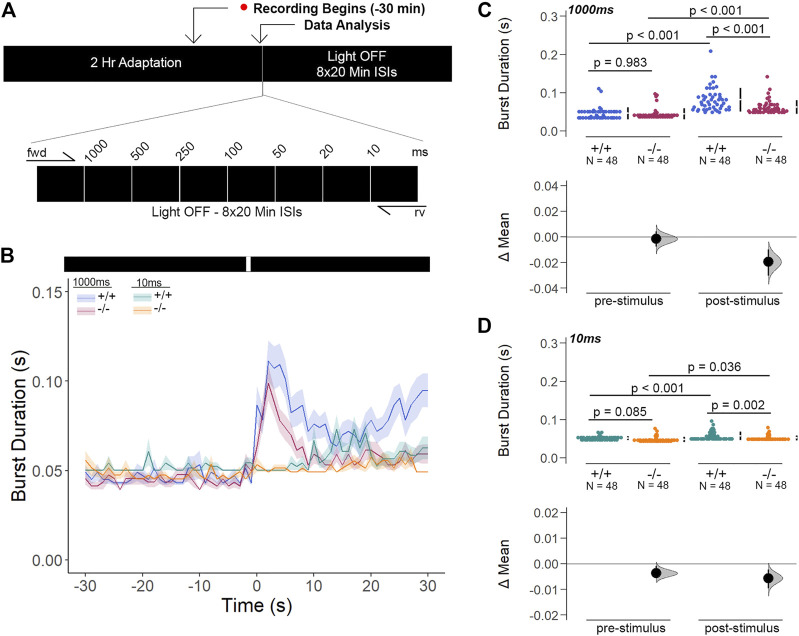
*Gjd2b* ^*−/−*^/Cx35.1^−/−^ Larvae Have Normal Threshold Responses to Flash Stimuli. **(A)** Overview of the FSTR assay protocol. Stimuli were presented in both the forward (fwd) and reverse (rv) direction to eliminate attenuation due to habituation. **(B)** Dark-adapted larvae were subject to light stimuli of various lengths. Average responses within 30s of the **(C)** 1000 m and **(D)** 10 m stimuli were plotted. **(C, D)** The mechanisms underlying visual processing speed was functionally intact in *gjd2b* ^*−/−*^ */*Cx35.1^−/−^ larvae as determined by a positive FSTR response. The statistical significance was determined by a Two-Way ANOVA test, *p* ≤ 0.05. The sample size (N) was 48 for WT and *gjd2b* ^−/−^ */*Cx35.1^−/−^ larvae.

Next, we inquired whether the light-ON VMR hyperactivity of *gjd2b/Cx35.1*-deficient larvae was caused by rapid temporal processing of visual stimuli. To determine the changes in visual processing speed, we developed the flash stimuli threshold response (FSTR) assay where the responsiveness of dark-adapted zebrafish was measured with transient light flashes at several different exposure times (experimental endpoints 1000 m and 10 m were sufficient for analysis). The FSTR was resolved by calculating the burst duration 30s before and after light stimulation and determining whether there was a change in response (Figures 8A,B). No change in FSTR responses meant that larvae were unable to discriminate the transient light stimulus, indicative of a slower processing speed, whereas an increased FSTR response indicated that the innate processing speed was functionally intact. The burst duration of both genotypes in response to a 1000 m light flash was significantly increased after light stimulation (in s, WT: 0.082 ± 0.04 × 10^−1^, *p* < 0.001, Cx35.1^−/−^: 0.062 ± 0.03 × 10^−1^, *p* < 0.001)*,* but was indistinguishable prior to the visual stimuli (in s, WT: 0.046 ± 0.02 × 10^−1^, Cx35.1^−/−^: 0.045 ± 0.02 × 10^−1^, n = 48, *p* = 0.983, *Two-Way ANOVA*). In contrast to light-ON VMR, *gjd2b* ^*−/−*^ */*Cx35.1^−/−^ larval responses were attenuated following the 1000 m light flash; the burst activity increased by 178% for the WT, but only 138% for *gjd2b* ^*−/−*^ */*Cx35.1^−/−^ larvae (*p* < 0.001) (Figure 8C). Similar results were obtained when larvae were exposed to a short 10 m light flash. Prior to the light stimulus, the burst activity of both genotypes was indistinguishable (in s, WT: 0.052 ± 0.01 × 10^−1^, Cx35.1^−/−^: 0.048 ± 0.01 × 10^−1^, *p* = 0.085). Upon stimulation with 10 m light-ON, WT and *gjd2b* ^*−/−*^ */*Cx35.1^−/−^ larvae increased their average burst duration by 112% and 109% respectively (in *s*, WT: 0.058 ± 0.02 × 10^−1^, *p* < 0.001, Cx35.1^−/−^: 0.052 ± 0.01 × 10^−1^, n = 96, *p* = 0.036). The *gjd2b* ^*−/−*^ */*Cx35.1^−/−^ responses following the 10 m flash stimulus were again reduced in comparison to the WT (*p* = 0.002) (Figure 8D). As such, we concluded that the hyperactivity to light-ON VMR in the *gjd2b* ^*−/−*^/Cx35.1^−/−^ larvae was not caused by a fast-processing mechanisms. Instead, we found that the short abrupt changes in light intensity caused *gjd2b* ^*−/−*^/Cx35.1^−/−^ hypoactivity in comparison to the WT; whereas long abrupt changes, such as those performed during VMR assay, had the opposite effect. Nevertheless, the ability to respond to the visual stimulus appeared intact in *gjd2b* ^*−/−*^ */*Cx35.1^−/−^ larvae, but to varying degrees.

### 3.8 Rod photoreceptor recruitment reverses light-ON hyperactivity in *gjd2b* ^*−/−*^/Cx35.1^−/−^ larvae

**FIGURE 9:**
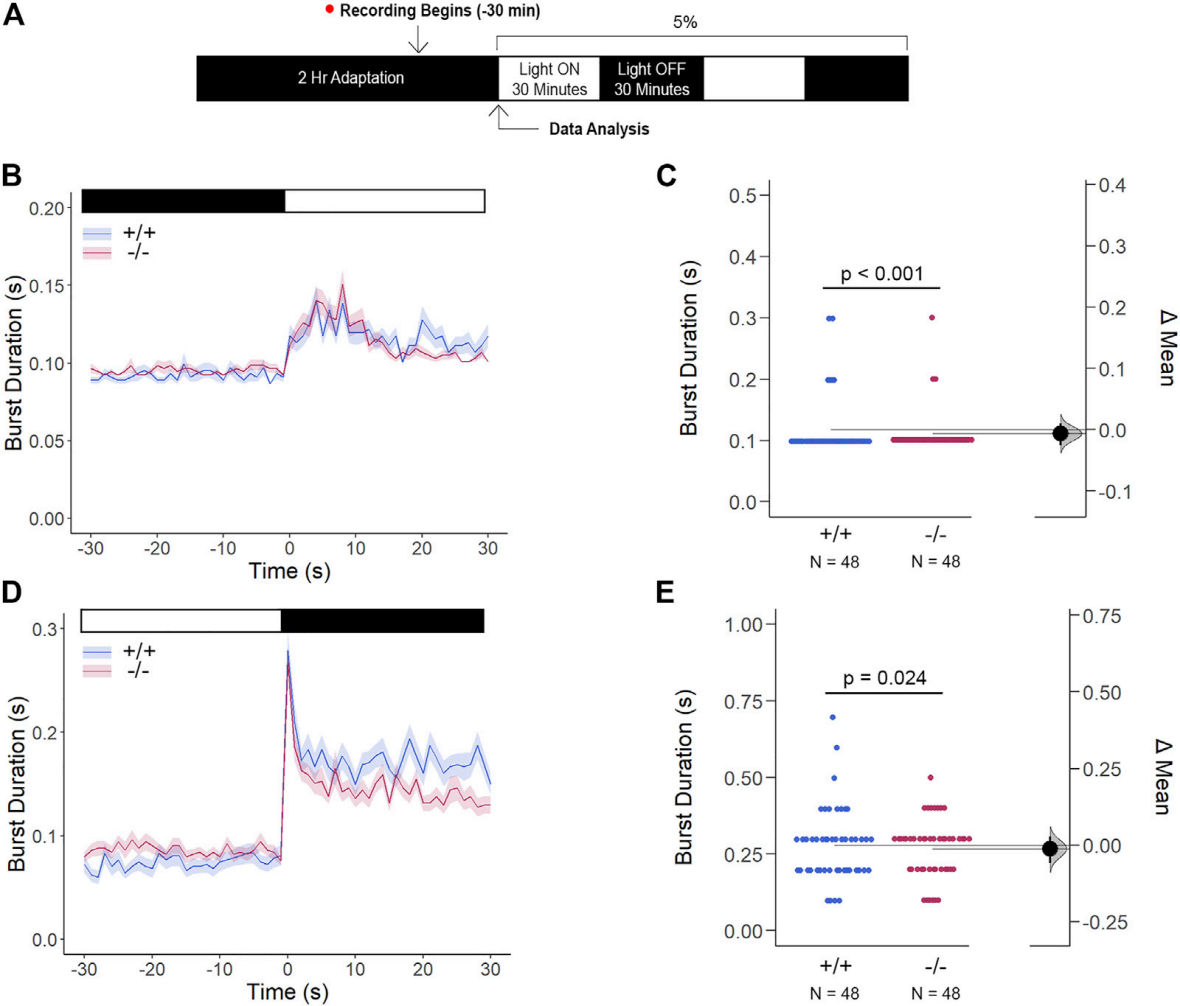
*Gjd2b* ^*−/−*^/Cx35.1^−/−^ Light-ON VMR Hyperactivity is Reversed with Rod Photoreceptor Recruitment *via* Mesopic Light Exposure. **(A)** General overview of the mesopic VMR Assay. Zebrafish larvae were exposed to 5% light intensity to determine their sensitivity to mesopic vision. **(B–C)** Light-ON responses revealed that the recruitment of rod-PRC activity reversed the burst hyperactivity previously seen in *gjd2b* ^*−/−*^/Cx35.1^−/−^ Light-ON VMR responses. **(D–E)** Light-OFF responses were reduced in *gjd2b* ^*−/−*^/Cx35.1^−/−^ larvae. The statistical significance was determined by the Mann-Whitney test, *p* ≤ 0.05. The sample size (N) was 48 for WT and *gjd2b* ^*−/−*^/Cx35.1^−/−^ larvae.

Li *et al.* have previously demonstrated that Cx35 electrical coupling facilitates both cone-cone and rod-cone photoreceptor crosstalk, the latter of which allows for saturated rod signaling to reroute to cones when exposed to the mesopic visual range (Li et al., 2009). Here, we determined how Cx35.1 expression, or lack thereof, influenced the visual responsiveness mediated by rod-cone coupling by performing a modified VMR test with mesopic light conditions after dark-adaptation (Figure 9A). In this test, *gjd2b* ^*−/−*^ */*Cx35.1^−/−^ larvae were marginally, yet significantly, hypoactive in comparison to the WT when exposed to the light-ON transitions (in s, 5% light-ON - WT: 0.117 ± 0.007, Cx35.1^−/−^: 0.111 ± 0.005, n = 48, *p* < 0.001) (Figures 9B,C). Similar results were found during the light-OFF VMR (in s, WT: 0.279 ± 0.017, Cx35.1^−/−^: 0.267 ± 0.014, *p* = 0.024) (Figures 9D,E). Taken together, our results suggested that low light conditions were sufficient to reverse light-ON hyperactivity.

### 3.9 Loss *of gjd2b*/Cx35.1 does not alter cone opsins expression or the innate color preference

The behavioral studies were complemented with a quantification of opsin and rhodopsin gene expression. Our own RNA-seq analysis had demonstrated that 6dpf TL larvae express red (*opn1lw2*), green (*opn1mw1* and *opn1mw2*), ultraviolet (*opn1sw1*), blue (*opn1sw2*), low-light rhodopsin (*rho*), and the second rhodopsin like (*rhol*) photopigments (GEO database entry GSE181853 (Whyte-Fagundes et al., 2022)). The qRT-PCR analysis showed an upregulation of *opn1mw2* expression (1.394-fold, *p* = 0.018), whereas the remaining cone transcripts were indistinguishable from the WT (Supplementary Figure S4, Supplementary Table S5). The observed downregulation of the rod photopigment *rho* (0.477-fold, *p* < 0.001) and *rhol* (0.727, *p* = 0.012) was in line with the behavioral changes to low light.

**FIGURE 10:**
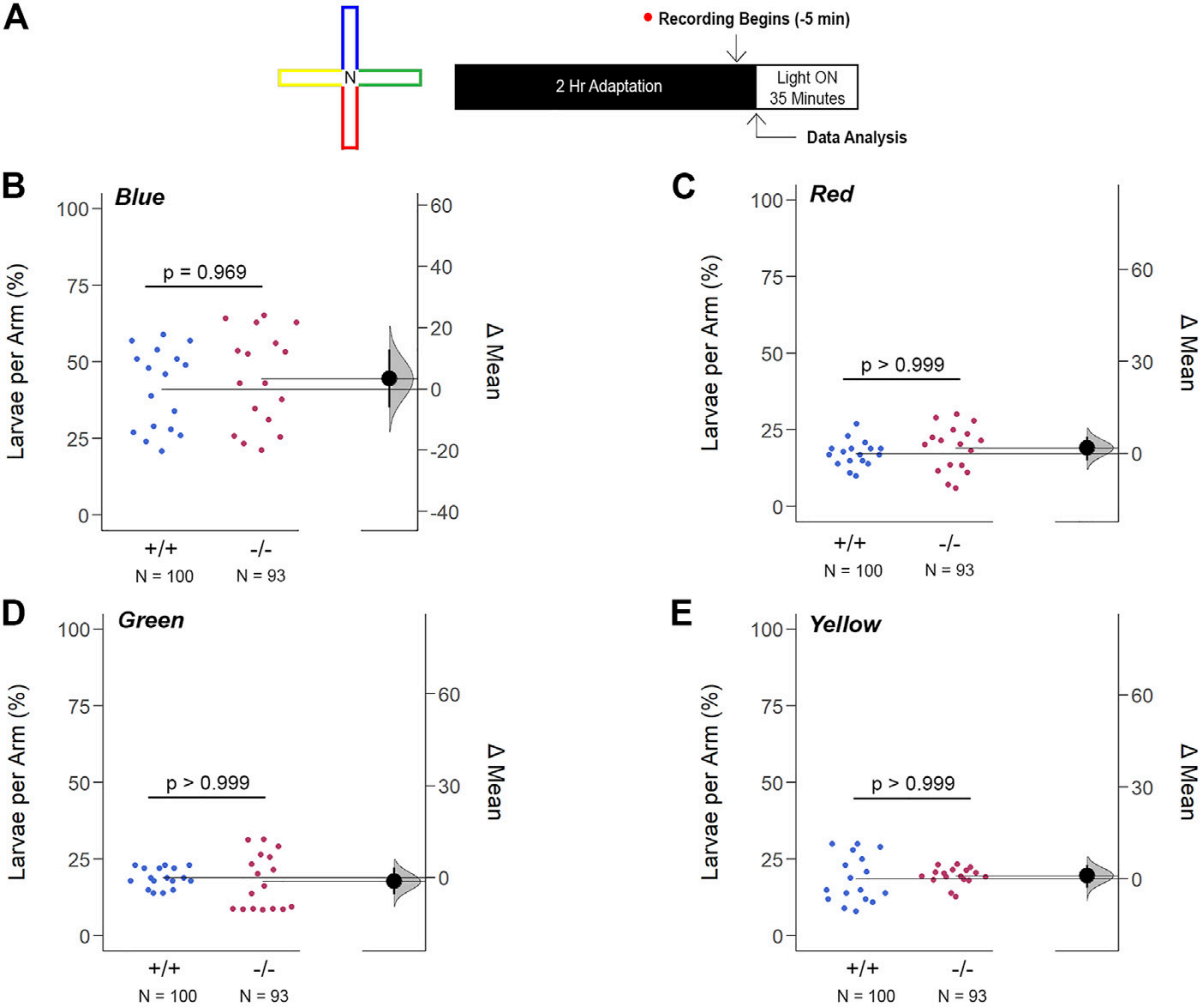
Loss of *gjd2b*/Cx35.1 Does Not Alter the Innate Color Preference. **(A)** Overview of the experimental cross-maze and protocol design used to assess innate color preference. The center of the cross maze was devoid of color and denoted as ‘N’ for neutral **(B–E)** Summary of the mean larvae per arm. Both genotypes equally preferred the blue arm; however, all else were indistinguishable. The statistical significance was determined by a Two-Way ANOVA test, *p* ≤ 0.05. The sample size (N) was 100 for WT and 93 for *gjd2b* ^*−/−*^/Cx35.1^−/−^ larvae.

The innate color preference test of 6dpf larvae confirmed that the increase in *opn1mw2* expression in *gjd2b* ^*−/−*^ */*Cx35.1^−/−^ larvae was insufficient to impact color vision prominently. Using a cross-maze described previously (Park et al., 2016), the experimental design accounted for potential location bias by implementing a dark adaptation period to confirm an equal distribution throughout the maze prior to the recording stage and by repetitions in which the colors within each arm of the maze were rearranged (Figure 10A). Consistent with previous reports (Avdesh et al., 2012; Park et al., 2016), WT larvae preferred blue (in %, 41.18 ± 2.13, n = 100, *p* < 0.001, *two-way ANOVA*). However, we found that the WT were indifferent towards to the red, green, and yellow colors (in %, Red: 17.35 ± 0.88, Green: 18.94 ± 1.83, Yellow: 18.53 ± 1.43, *p* > 0.999). Similarly, *gjd2b* ^*−/−*^ */*Cx35.1^−/−^ larvae preferred blue (in %, 44.55 ± 2.71, n = 93, *p* < 0.001) with no distinguishable preference for the other colors (in %, Red: 19.06 ± 1.10, Green: 17.73 ± 1.25, Yellow: 19.56 ± 0.96, *p* > 0.999) (Supplementary Figure S5). No statistically robust difference in the color preference was detected between the two genotypes (Blue: *p* = 0.969, Red: *p* > 0.999, Green: *p* > 0.999, Yellow: *p* > 0.999) (Figures 10B–E).

Next, we modified the color preference test and restricted the color choice to two colors and a neutral choice. Here, the blue color remained attractive when compared to a neutral stimulus (in %, Blue: 50.50 ± 1.54, Neutral: 44.83 ± 1.67, n = 40; *p* = 0.011) (Supplementary Figure S6). In any other combination of green, red, and yellow stimuli, larvae of both genotypes preferred the neutral stimulus. All the above suggested that Cx35.1 depletion did not alter color vision despite the upregulation of *opn1mw2* expression.

## 4 Discussion

In this study, we aimed to provide insight into the potential roles of *gjd2b/*Cx35.1 in the developing visual system of zebrafish larvae. We describe a new mutant line generated with Cas9/CRISPR technology, which differs from the *gjd2b* ^*fh454*^ (Miller et al., 2017) and *gjd2b* ^*ncb215*^ (Sitaraman et al., 2021) mutant lines generated by TALEN technology. The general strategy to obtain the three lines was similar; all targeted exon one of the *gjd2b* gene close to the start codon. A difference was the use of multiple zebrafish strains; the transgenic line *M/CoLo:*GFP (*Et* (*Tol-056:GFP*)) with an AB/Tu (Miller et al., 2017), Indian WT (Sitaraman et al., 2021), or TL background in this study. Another difference is the minimal 1bp substitution (position G12 →A) altering amino acid 4 (tryptophan 4) to an early stop codon (after amino acid 3) in our study. In contrast, the *gjd2b* ^*ncb215*^ line has an insertion after the translation start site causing a frame shift and insertion of a premature stop codon, with a predicted truncated protein of 55 amino acid residues. How the different genetic backgrounds or the formation of a truncated protein contributed to phenotypic differences is presently unknown.

The depletion of Cx35.1 was investigated using an anti-Cx36 antibody (#37-4000; Thermofisher). This antibody was different to the anti-Cx35/36 antibody used previously to demonstrate Cx35.1 depletion (Quint et al., 2021; Sitaraman et al., 2021). Here, the immunoreactivity found in the photoreceptor and inner plexiform layers of the zebrafish retina at 6dpf was comparable to the rodent retina (Li et al., 2008) and previous reports showing staining of the neuropil in the optic tectum of larvae (Jabeen and Thirumalai, 2013), and at adult stages of the zebrafish (Li et al., 2009; 2014). Further, the observed Cx36 immunoreactivity overlapped with the single cell RNA sequencing data from Quint et al. (2021), which showed expression in all neuron subtypes (Quint et al., 2021). Consistent with the introduction of an in-frame translation stop signal, the Cx36 immunoreactivity was diminished in *gjd2b*/Cx35.1 KOs with residual fluorescence attributed to other Cx36 orthologs. The characterization of the mutant line suggested that the loss of Cx35.1 function led to various morphological phenotypes, including biometrical changes to the eyes and head. The molecular changes were consequential, affecting connexin gene expression, two signaling pathways, and behavioral outcomes in response to temporal and luminescence changes of visual stimuli.

### 4.1 *Gjd2b/*Cx35.1 uncoupling causes biometrical changes to eyes and head

In humans, *GJD2* (Cx36) is associated with refractive error (Kunceviciene et al., 2022). As discussed previously by Quint et al. (2021) two common genetic variants downstream of *GJD2* (Cx36) have consistently been associated with refractive error (rs634990 and rs524952) and myopia. However, the coding region of *GJD2* (Cx36) has not revealed coding variants explaining the genome wide association study results; it has been suggested that the identified variants might play a role through gene regulation. As pointed out by Quint et al. (2021) the identified risk variants for myopia might upregulate the expression of *GJD2*, facilitating the coupling of Cx36 (*GJD2*) gap junctions in the eye. Following this idea, the uncoupling of gap junctions could explain the opposite outcome.

In our study the Cx35.1 depletion caused no cataracts when the genetic background was TL. This phenotype differs from Quint et al. (2021) which describes lenticular cataract formation as the main outcome of Cx35.1 depletion. Instead, loss of Cx35.5 caused a hyperopic phenotype when the genetic background was AB. We do not disagree with the principal results of Quint et al. but like to point out that previously reported RNA-seq and the qRT-PCR results shown in this study demonstrate a robust expression of *gjd2b/*Cx35.1 in the TL strain. The robust expression of *gjd2b*/Cx35.1 is consistent with the reported single-cell RNA-seq data showing the expression of this gene in all major cell types of the developing retina. Interestingly, we found that *gjd2b*/Cx35.1 depletion caused a downregulation of *gjd1b*/Cx34.7. Previous work has demonstrated that both genes are expressed in cone photoreceptors (O'Brien et al., 1998; O'Brien et al., 2004). G*jd2b*/Cx35.1 and *gjd1b*/Cx34.7 form heteromeric gap junctions with asymmetric function in mixed electrochemical synapses (Rash et al., 2013; Rash et al., 2015). The concurrent depletion of the two connexins adds to the accumulating evidence for the molecular and functional asymmetry of electrical synapses (O'Brien, 2014; Palacios-Prado et al., 2014).

### 4.2 The molecular profile of *gjd2b* ^*−/−*^/Cx35.1^−/−^ larvae favors alternative junctional coupling mechanisms

The molecular mechanisms explaining the relationship between Cx35.1 mediated gap junction uncoupling and the observed changes to eye growth and head abnormalities are unknown. However, our results provide the first evidence that links Cx35.1 depletion with the Wnt/ß-catenin signaling pathway. Wnt/ß-catenin signaling is known to affect brain development and the formation of camera eyes at embryonic stages. Genome-wide association studies in humans have identified loci causing refractive errors which are linked to Wnt signaling (Cheng et al., 2013). In the zebrafish we identified a downregulation of *wnt8b*, *wnt11*, and the receptor *fdz5* when *gjd2b*/Cx35.1 was lost. *Wnt8a* and *wnt8b* are required to activate the Wnt/ß-catenin signaling pathway and the integration of *wnt11*, *fdz5*, and Wnt/β-catenin signaling coordinates cell fate determination and morphogenesis of the developing brain (Cavodeassi et al., 2005). *Wnt8b* has roles in biological processes like eye, forebrain and hypothalamus development (Kim et al., 2002; Lee et al., 2006), and neuronal differentiation, including the cluster size of dopaminergic neurons (Russek-Blum et al., 2008). We propose that the Cx35.1 KO models generated in different research programs are assets for in-depth investigations into how Cx35.1 modulates Wnt/β-catenin signaling through gap junction (un)coupling during development and maturation.

Another point of interest in Cx35.1-deficient larvae was motivated by the regulation of the D2-receptor genes *drd3* and *drd4b*, and *vmat2* (alternative gene name: *slc18a2*). In mammals, five distinct types of dopamine receptors have been identified and are classified into two families; D1-like (D1R and D5R), which are stimulatory subsets to adenyl cyclase, and D2-like (D2R, D3R, and D4R) which inhibit adenyl cyclase (Girault and Greengard, 2004). D4R subtypes are expressed in rod and cone PRCs of the outer retina of different vertebrates (Alfinito and Townes-Anderson, 2001; Ostergaard et al., 2007; Ma et al., 2013), and drd3 gene transcripts have been found in teleost retinal ganglion cells (Boehmler et al., 2013). Literature provides well-described evidence of similar expression patterns for D1-like and D2-like subtypes across the retina of different vertebrates (Callier et al., 2003; Yamamoto et al., 2013). Typically, photopic conditions increase dopamine production (Witkovsky, 2004) and Vmat2 plays a role in vesicular dopamine transport (Esposito et al., 2012). PRC coupling has been linked to dopamine and the D4R subtype (Li et al., 2013; O'Brien et al., 2004). Li et al. (2014) has concluded that adenosine and dopamine coregulate photoreceptor coupling through opposite action on the protein kinase A (PKA) pathway and Cx36 phosphorylation (Li et al., 2013). Therefore, under photopic light conditions, gap junctions will be primarily de-phosphorylated, resulting in the uncoupling of PRCs; this is critical in supporting high contrast vision. The opposite occurs under scotopic vision where PRCs are coupled in a phosphorylation-dependent manner (Kothmann et al., 2007; Kothmann et al., 2009; Li et al., 2009). Considering this, we propose that the reduction of *drd3* and *drd4b* receptor subtypes, together with the upregulation of *vmat2*, modulates the phosphorylation cascade. Although this provides evidence for a dopaminergic-dependent compensatory mechanism in Cx35.1^−/−^ larvae, it remains unclear which Cx36 ortholog(s) or other retinal connexins could be phosphorylated. An alternative explanation could derive from the functional antagonism of dopamine and adenosine receptors, specifically the A_2A_ and D2-like subtypes. In such circumstances, activation of A_2A_ receptors reduces in dopamine affinity (Franco et al., 2000). In this regard, the actions of A_2A_ receptors on the available D2-like receptors would complement proposed mechanism by reducing phosphorylated Cx36 orthologs in our *gjd2b* ^*−/−*^ */*Cx35.1^−/−^ model. However, the relative expression of A_2A_ was not explored in this study and deferred to follow up studies. Further, we detected a downregulation of two connexins associated with expression in the eye (*gja2*/Cx39.9, *gja3*/Cx48.5) (Zoidl et al., 2008). The Cx48.5 protein is involved in post-embryogenic eye development (Cheng et al., 2003) and the gja3^s213/s215^ and gja^s226/s226^ lines display a decreased eye size (Chi et al., 2008). Interestingly, experimental depletion of *gja3*/Cx48.5 with morpholinos caused cataracts, which we have not observed here. We conclude that the moderate downregulation detected in our fish model was insufficient to cause a similar phenotype. However, in mammalians and humans, the expression of mutants of the orthologous gene Cx50 is associated with cataract formation, smaller lenses, and eyes, indicating the importance of gap junctions in the maintenance of lens fiber homeostasis in mammals (Beyer and Berthoud, 2014; Berthoud et al., 2020; Retamal and Altenberg, 2022).

Hirata et al. (2012) showed that morpholino-based depletion of *gja2*/Cx39.9 in the zebrafish does not affect the locomotory behavior but caused abnormal thigmotaxis and changes to muscle contraction (Hirata et al., 2012). It remains to be determined how this gene contributes to the observed phenotype of our model.

### 4.3 *Gjd2b*/Cx35.1 un-coupling alters visually evoked behaviors

To gain insight into how *gjd2b/*Cx35.1-mediated gap junctions regulate brain functions, we focused on testing visually evoked behaviors based on a significant reduction in Cx36-immunoreactivity in the IPL and in a subpopulation of photoreceptor cells, which was attributed to the loss of Cx35.1. The IPL houses the synaptic connections between ganglion cells and amacrine and bipolar cells and is thought to be the region in which motion, brightness, contrast, and hue are processed (Wassle and Boycott, 1991; Calkins and Sterling, 1999). Pan et al. (2010) reported a partial or complete loss of ganglion-to-amacrine cell coupling in Cx36 knock-out mouse models concluding that Cx36 expression was critical in driving the connectivity between most ganglion cell subtypes (Pan et al., 2010). In our *gjd2b* ^*−/−*^ */*Cx35b^−/−^ larvae, the atypical pattern of fluorescence observed at the PRC layer suggests that *gjd2b/*Cx35.1 is localized to specific PRC subtype(s); the lack of access to a Cx35.1 specific antibody did not allow us to resolve this question. However, we also found Cx36 immunoreactivity in the optic nerve and the arborization fields (AF) where RGC axons contact dendrites of tectal neurons (Robles et al., 2013; Baier and Wullimann, 2021; Guggiana Nilo et al., 2021). Previous work also has demonstrated that Cx35.1 depletion altered dendrites and synaptic plasticity in Purkinje neurons of the zebrafish (Sitaraman et al., 2021). We reasoned that *gjd2b/*Cx35.1 could have similar roles in other brain regions and that altered dendrites and synaptic plasticity could affect processing of visual information by impairment of sensory integration and disturbances in motor outputs that manifest as measurable changes in locomotion and visual acuity.

Considering the *gjd2b/*Cx35 expression in the visual system previously noted (Kothmann et al., 2007; Marsh et al., 2017; Miller et al., 2017), and the changes observed in our knock-out model, we explored several visually guided locomotor behaviors. Firstly, we assessed their free swim activity and found that *gjd2b* ^*−/−*^ */*Cx35.1^−/−^ larvae were largely comparable to the wild-type. Due to the zebrafish’s innate avoidance of darkness (Mansur Bde et al., 2014), we anticipated a reduction in overall swimming activity in Light-OFF (dark) conditions regardless of genotype. We did note that *gjd2b* ^*−/−*^ */*Cx35.1^−/−^ larvae had a reduced duration of bout and glide behavior (defined as swim speeds >0 and <20 mm/s) that was exclusive to darkness, however, it is unclear whether this can be attributed to a motor-muscle deficiency, anxiety-like phenotype, or a combination of them both. Nevertheless, this served as our initial basis that processing of luminescence requires functional *gjd2b/*Cx35.1 expression.

The Visual Motor Response (VMR) assay (Emran et al., 2008; Liu et al., 2015) was used to better characterize how luminescence changes impacted the behavioral responses of larvae of both genotypes. The expectation that both rods and cone photoreceptor cells retained some functionality despite the loss of *gjd2b/*Cx35.1 was observed; *gjd2b* ^*−/−*^ */*Cx35.1^−/−^ larvae responded to sudden changes in luminescence, although this was met with an increased sensitivity to light-ON transitions. Under this condition, cone photoreceptor stimulation and subsequent downstream ON-bipolar cell activation is the primary signaling mechanism, providing evidence that *gjd2b/*Cx35.1 contributes to cone PRC activity. In support of this, exposing *gjd2b* ^*−/−*^ */*Cx35.1^−/−^ larvae to a mesopic visual range (5% light intensity) reversed the light-ON VMR hyperactivity, indicating that the recruitment of rod photoreceptor cells was able to reverse this response. Li et al. have previously demonstrated that Cx35 electrical coupling facilitates both cone-cone and rod-cone photoreceptor crosstalk, the latter of which allows for saturated rod signaling to reroute to cones when exposed to the mesopic visual range (Li et al., 2009). Taken together, our results support previous research regarding the role of Cx35.1 in mediating cone PRC coupling (Li et al., 2009). We propose that *gjd2b/*Cx35.1 mediates cone PRCs crosstalk with a primary role in fine-tuning communication between nerve cells. Since larvae had altered D2-receptor family and *vmat2* expression, we suspect that pre- and/or postsynaptic dopamine signaling may contribute to the observed cone-mediated sensitivity. However, this hypothesis requires further study of how changes in dopaminergic signaling alter cone functions and its similarity to the known effects on visual acuity following depletion of the retinal dopaminergic interplexiform cells (Li and Dowling, 2000).

### 4.4 *Gdj2b*/Cx35.1 contributes to a spatiotemporal processing but not chromaticity

An alternative explanation to light-ON VMR hyperactivity takes into consideration the upregulation of the opsin gene *op1mw2*. Berry et al. (2019) have found that viral-delivered medium-wave opsin restored visual sensitivity and speed in blind rd1 mice to allow for spatiotemporal discrimination under dim-light and room-light (photopic) conditions; a result rhodopsin was unable to achieve (Berry et al., 2019). As such, we cannot discount that *op1mw2* upregulation may have provided additional visual sensitivity, though we believe this is unlikely since we provide evidence that *gjd2b/*Cx35.1 uncoupling does not alter the spectral sensitivity. Previous reports have revealed the innate color preferences of adult zebrafish (Avdesh et al., 2012; Bault et al., 2015) and larvae as early as 5dpf (Park et al., 2016). Although the reported evidence for red, green, and yellow color preferences is inconsistent in the literature, zebrafish larvae in this study consistently presented a robust preference for blue. The preference was identical in both genotypes, which led us to conclude that the differential expression of the green cone opsin gene *op1mw2* in *gjd2b/*Cx35.1^−/−^ larvae was insufficient to impact innate color preferences and thus spectral sensitivity.

The quality of visual processing can be quantified by the measurement of luminescence, chromaticity, and spatiotemporal properties of light stimuli. Our VMR studies established roles of *gjd2b/*Cx35.1 in the discrimination of luminescence and the color preference test addressed chromaticity; to overcome the limited information regarding spatiotemporal properties, the FSTR assay was performed. Here, our results revealed that *gjd2b* ^*−/−*^ */*Cx35.1^−/−^ larvae responded to stimuli short in duration (10 m). In support of this result, Quint et al. (2021) demonstrated that electroretinograms of *gjd2b* ^*−/−*^ */*Cx35.1^−/−^ measuring the B-wave responses in zebrafish were indistinguishable from the wildtype (Quint et al., 2021). Though we concluded that the visual processing speed remained intact in *gjd2b* ^*−/−*^ */*Cx35.1^−/−^ larvae, the overall behavioral responses were more robust during VMR (i.e., with longer durations of light stimulation) than during FSTR. As such, this revealed an unexpected contribution of *gjd2b/*Cx35.1 expression to vision in mediating behavior based on the temporal features of the stimuli.

This study found that *gjd2b*/Cx35.1 depletion leads to changes in the refractive properties of the eye, leading to hyperopic shifts, and morphological changes to the head and body ratio of zebrafish larvae. The changes to Wnt/ß-catenin and dopaminergic signaling pathways, as well as retinal immunostainings, identify novel roles for *gjd2b/*Cx35.1. As such, the results provide evidence for the contribution of *gjd2b*/Cx35.1 in the development of the visual system and visually guided behaviors in zebrafish. To our knowledge, we are the first to describe these distinct behavioral responses in *gjd2b/*Cx35.1 deficient zebrafish. While the details surrounding junctional coupling in *gjd2b* ^*−/−*^ */*Cx35.1^−/−^ zebrafish remain unclear at present, we speculate that they are caused at the synaptic level by changes to the plasticity and functional asymmetry of electrical synapses. In conclusion, the evidence provided from phenotypic, molecular, and behavioral analysis support that significant functions of *gjd2b/*Cx35.1 in visual processing through the differential discrimination of luminescence and temporal properties, mediated by cone photoreceptors, start at the early stages of development.

## Data Availability

The original contributions presented in the study are included in the article/Supplementary Material, further inquiries can be directed to the corresponding authors.
