# Supplementary material for: Gap junction Delta-2b (gjd2b/Cx35.1) depletion causes hyperopia and visual-motor deficiencies in the zebrafish
Source: Front Cell Dev Biol. 2023 Mar 2;11:1150273. doi: 10.3389/fcell.2023.1150273 (PMC10017553; doi:10.3389/fcell.2023.1150273)
Supplement: Supplementary file 1 [file DataSheet1.docx]

Supplementary Material

Gap Junction Delta-2b (*gjd2b*/Cx35.1) Depletion Causes Hyperopia and Visual-Motor Deficiencies in Zebrafish

Cherie A. Brown^1,4*^, Shiva S.R. Aghdam^3^, Georg S.O. Zoidl^1,4^, Christiane Zoidl^1,4^, Nima Tabatabaei^3,4^, Georg R. Zoidl^1,2,4*^

^1^ Department of Biology, York University, Toronto, Ontario, Canada

^2^ Department of Psychology, York University, Toronto, Ontario, Canada

^3^ Department of Mechanical Engineering, York University, Toronto, Ontario, Canada

^4^ Center for Vision Research, York University, Toronto, Ontario, Canada

*** Correspondence:**Cherie A. Brown, cherie.brown@ucalgary.ca; Georg R. Zoidl, gzoidl@yorku.ca

# Supplementary Figures


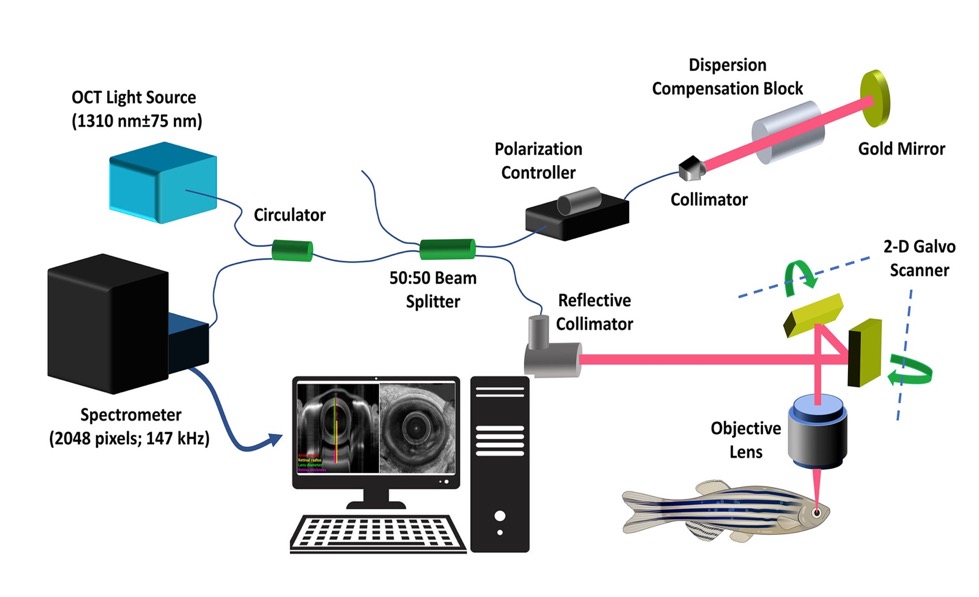


Supplementary Fig. 1: *Graphical representation of the* *custom-made spectral-domain optical coherence tomography (SD-OCT) system used for tomographic imaging of adult zebrafish.* The system utilized a 1310nm near-infrared super-luminescent diode light source (Exalos; Switzerland) to take images of zebrafish. A 50/50 fiber coupler was used to split light into the reference and the sample arms. To adjust polarization to the cross-polarization state a polarization controller was used in the reference arm. The SD-OCT system employed a line scan camera (2048 pixels with 140 kHz maximum line rate) in the spectrometer. A GPU (Graphics Processing Unit)-based processing program was developed for real-time display of OCT B-scans.


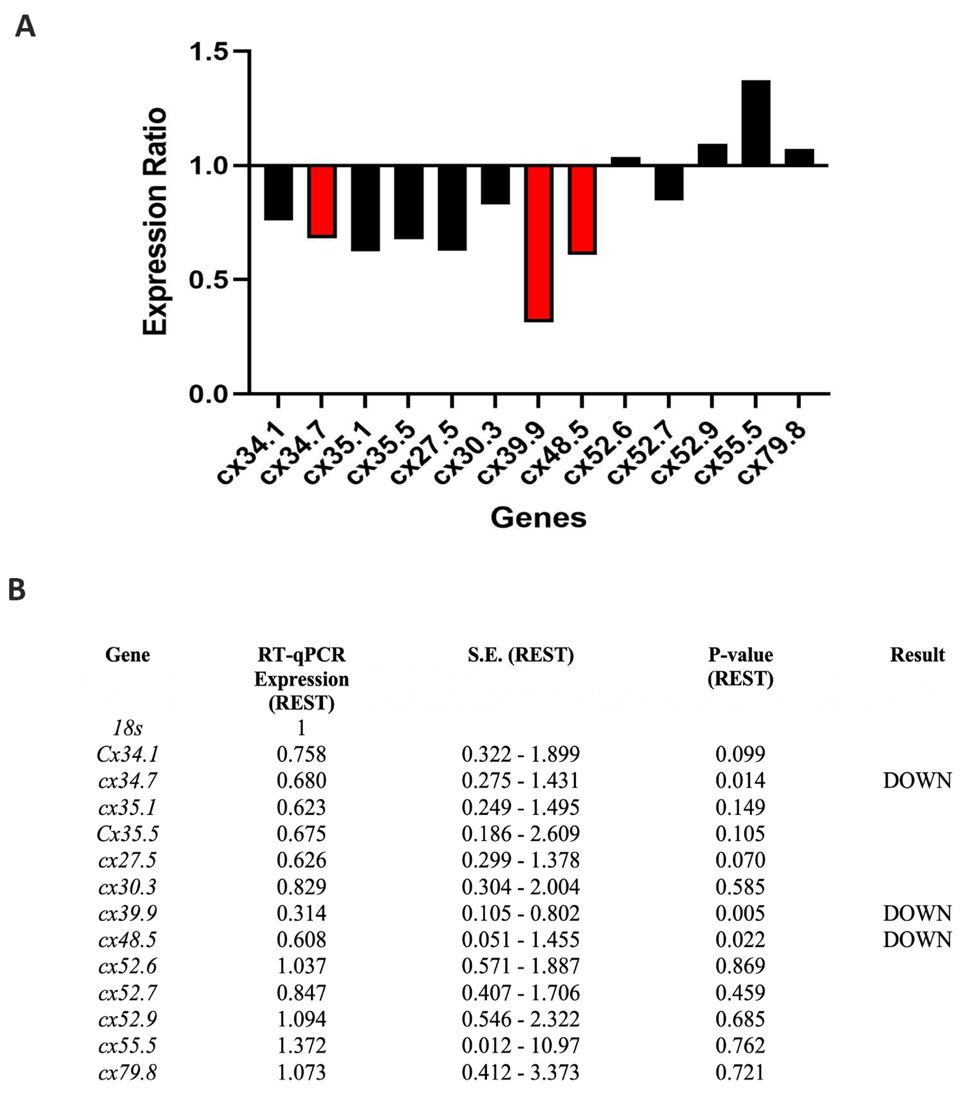


Supplementary Fig. 2: *Differential Expression of Connexin Genes in 6dpf Larvae.* A) Graphical presentation of relative expression ratios. B) Values were calculated using REST (Pfaffl et al., 2003). N = 4 replicates. S.E., standard error.


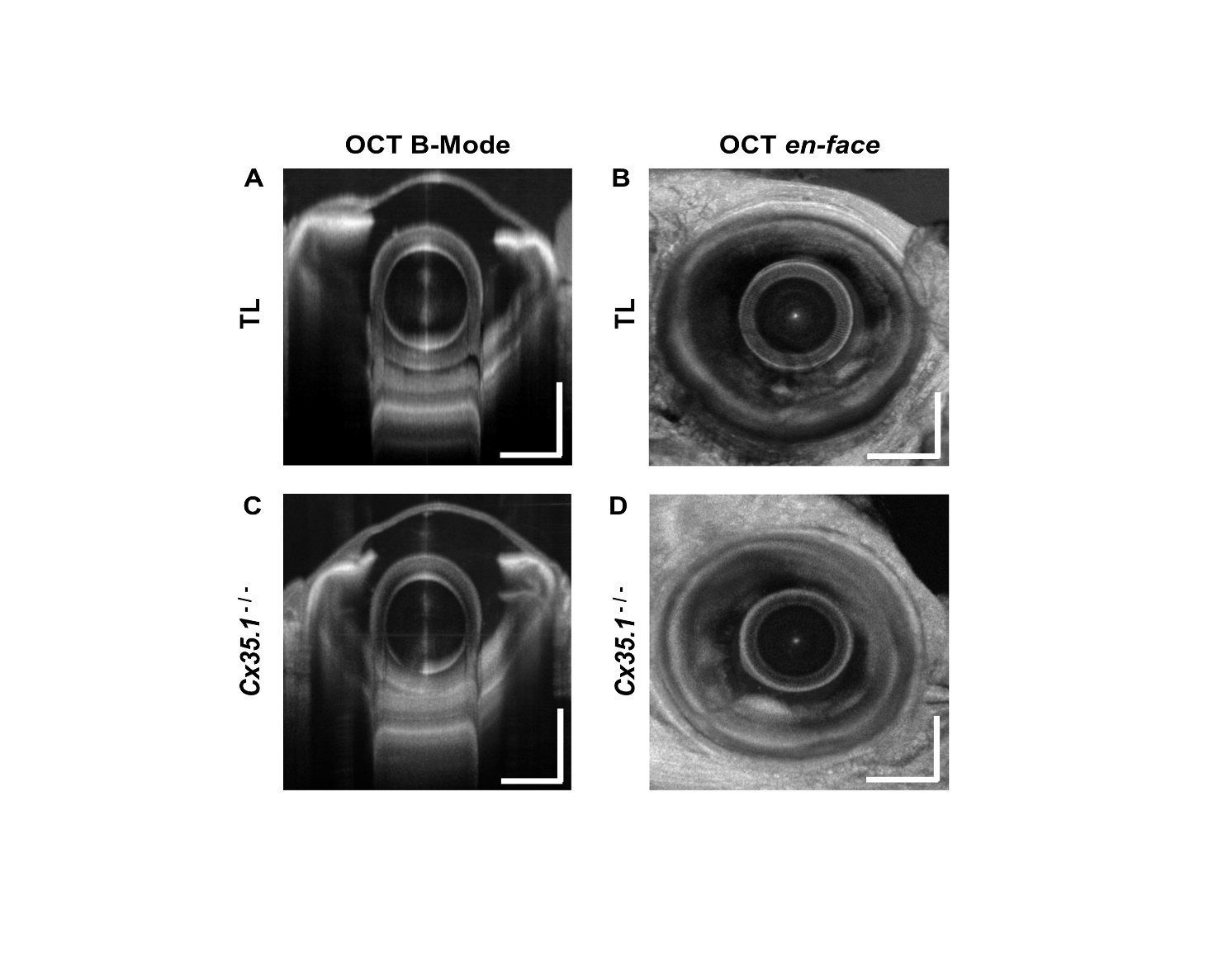


Supplementary Fig. 3: *Representative OCT images of adult WT and gjd2b^-/-^/*Cx35.1^-/-^ *eyes*. A,C show B-mode views. B,D show the en-face perspective. Scale bars = 500µm.


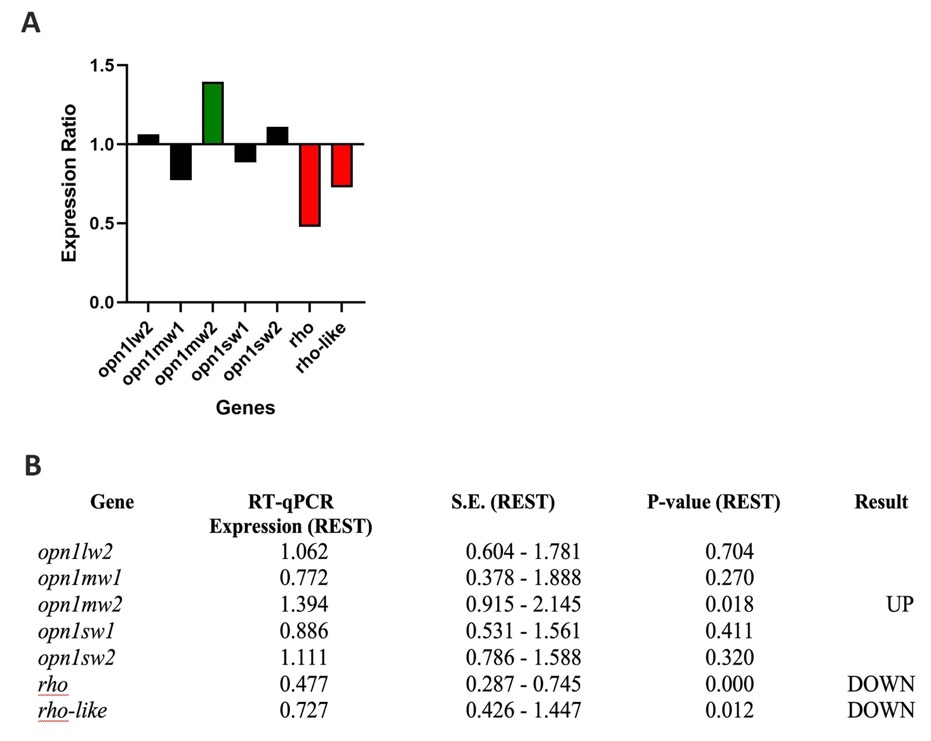


Supplementary Fig. 4: *Differential Expression of Connexin Opsin and Rhodopsin Genes in 6dpf Larvae.* A) Graphical presentation of relative expression ratios. B) Values were calculated using REST (Pfaffl et al., 2003). N = 4 replicates. S.E., standard error.


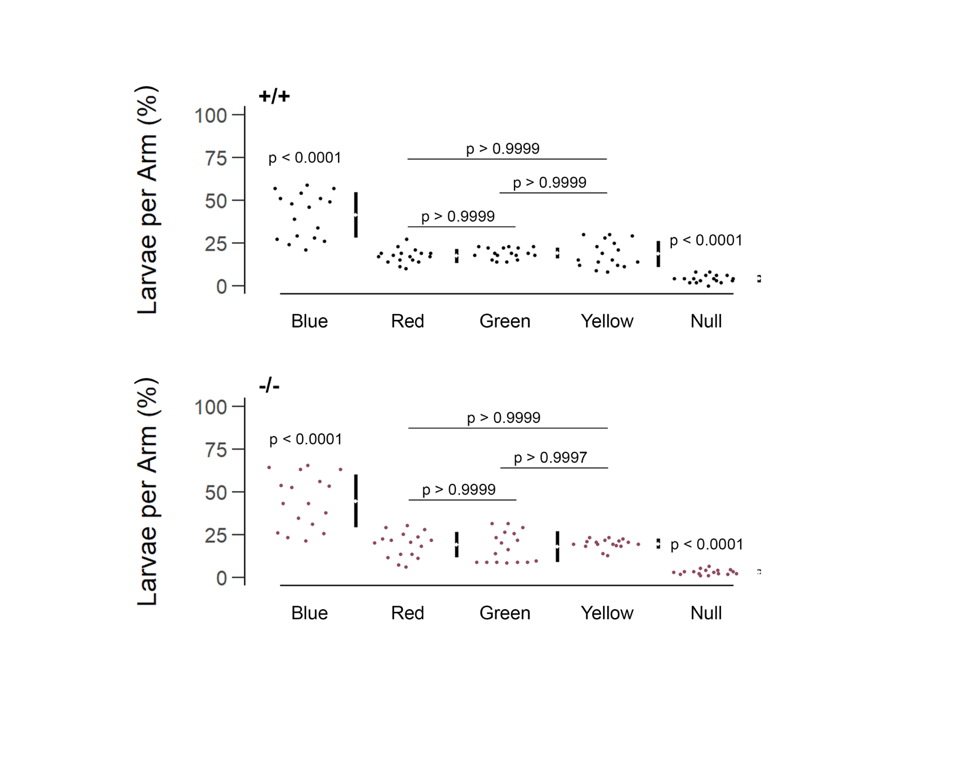


Supplementary Fig. 5: *Loss of gjd2b/*Cx35.1 *does not alter the innate color preference.* Summary of the mean larvae per arm. Both genotypes equally preferred the blue arm; however, all else were indistinguishable. The statistical significance was determined by a Two-Way ANOVA test, The sample size (n) was 100 for WT and 93 for *gjd2b^-/-^*/Cx35.1^-/-^ larvae.


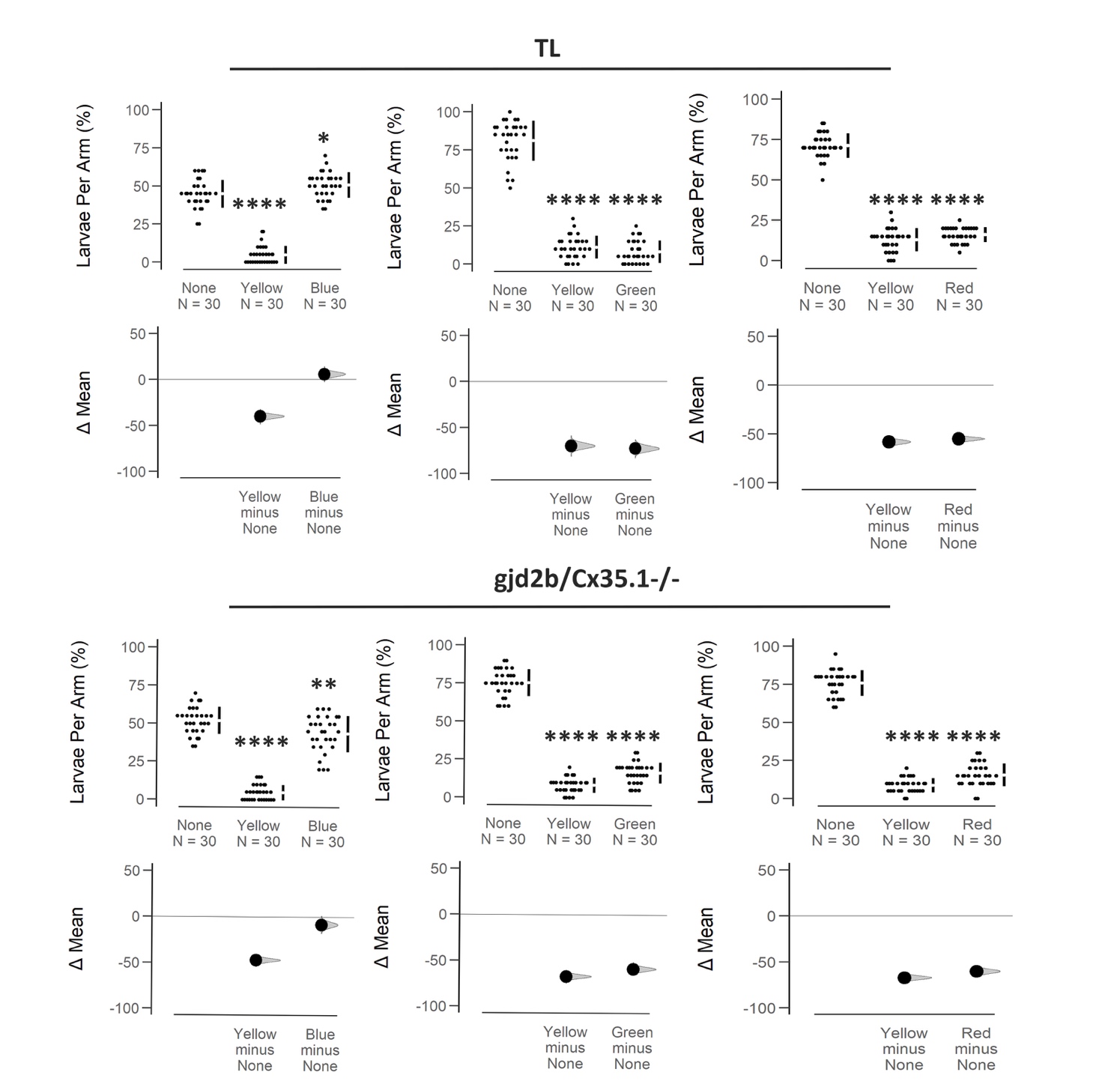


Supplementary Fig. 6: *2-arm innate color preference test.* 40 larvae were tested for each genotype. The statistical analysis used a One-way Anova with a Dunnet’s posthoc test. * p<0.05, * p<0.01; **** p<0.001.

# Supplementary Tables

Supplementary Table 1: qRT-PCR primers for analysis of connexin expression in *gjd2b*/Cx35.1^-/-^ larvae.

| Gene | Gene-ID | Forward Primer (5‘ – 3‘) | Reverse Primer (5‘ – 3‘) | Amplicon (bp) |
| --- | --- | --- | --- | --- |
| *18s** | NM_001098396 | tgcatggccgttcttagttg | agtctcgttcgttatcggaatga | 86 |
| *actb2** | NM_181601 | gcccctagcacaatgaagatc | gactcatcgtactcctgcttg | 134 |
| *tuba1b** | NM_194388 | gagcgtcctacttacaccaac | agggaagtggatacgaggatag | 146 |
| *cx27.5* | NM_131811 | actccaaagattccgctgtg | aggaactttacccaacacgg | 142 |
| *cx30.3* | NM_212825 | tctttccagtctcgcacatac | ctcctttaccattacctccacg | 150 |
| *cx34.7* | NM_001128766 | gccttcccaatctcccatatc | gtgtaacgctggtctttgtg | 122 |
| *cx35b* | NM_194420 | actgtgttcctcgtcttcatg | agcccaggtgattgagttc | 79 |
| *cx39.9* | NM_212826 | gcccaggaacattctacagtg | ctgagcaccaagatacggaag | 74 |
| *cx52.6* | NM_212819 | agagtgattcggtggaaagtg | ccccttgagtgatacggttg | 141 |
| *cx52.7* | NM_001113502 | gaccatcctgttcattttccg | tgagcgatattgggaatgcc | 146 |
| *cx52.9* | NM_207093 | caccatcctcttcatcttccg | agtgttgcagatgaagtcgg | 91 |
| *cx55.5* | NM_131812 | tttccccatttccctaatccg | gttctcgtctcagtgccatc | 146 |
|  |  |  |  |  |
|  |  |  |  |  |

* Indicates reference genes used in qRT-PCR

Supplementary Table 2. Quantification and statistical analysis of geometrical eye parameters extracted from OCT tomograms (n= 26/genotype).

| Parameter (unit) | TL (mean±STD) | Cx35.1^-/-^ (mean±STD) | P-value |
| --- | --- | --- | --- |
| Body length (mm) | 24.33 ± 1.84 | 24.94 ± 1.52 | 0.194 |
| Weight (g) | 0.27± 0.08 | 0.29 ± 0.05 | 0.358 |
| Lens ratio (µm/µm) | 1.01±0.02 | 1.02±0.02 | 0.150 |
| Retinal thickness (µm) | 196.07±15.17 | 204.16±25.36 | 0.169 |
| Axial/body length (µm/mm) | 8.92±0.86 | 7.97±0.50 | 0.000 |
| Lens radius (µm) | 362.12±20.50 | 351.15 ±17.99 | 0.046 |
| Retinal radius (µm) | 717.52±66.23 | 643.52±78.40 | 0.001 |
| RRE (µm/ µm) | 0.19±0.08 | 0.26±0.08 | 0.006 |

Supplementary Table 3: qRT-PCR primer for the analysis of dopamine-pathway genes.

| Gene | Gene-ID | forward primer (5‘ – 3‘) | reverse primer (5‘ – 3‘) | Amplicon (bp) |
| --- | --- | --- | --- | --- |
| *drd1a* | XM_017359120 | ctcatctccttcatcccagtg | atatgttcggttgaggctgg | 126 |
| *drd2a* | NM_183068 | atcgggatgggtgcatttc | tggtactccggaaaagacg | 101 |
| *drd2b* | NM_197936 | acctccaagtcccaatcatg | gttcgggttttgccattagg | 134 |
| *drd2c* | AY333792 | acctccaagtcccaatcatg | gttcgggttttgccattagg | 147 |
| *drd3* | NM_183067 | atcagtatcgacaggtatacagc | ccaaacagtagagggcagg | 137 |
| *drd4a* | NM_001012616 | cttaccgctgtttgtgtatgc | atgaacctgtctatgctgatgg | 144 |
| *drd4b* | NM_001012618 | ttacccctgtttgtctatgctg | ggctataaacctgtccacactg | 147 |
| *th* | NM_001001829 | ttgtgtccgagagctttgag | aagcattctggatcttggagg | 128 |
| *dat* | NM_131755 | gaatcgacagtgctatgggag | cagatgagcgagatgaggaag | 137 |
| *vmat2* | NM_001256225 | agctccttttcttatcctggc | tgcaatgaggatgtatgggtc | 141 |
|  |  |  |  |  |
|  |  |  |  |  |

Supplementary Table 4: qRT-PCR primer for the analysis of Wnt/frizzled pathway genes.

| Gene | Gene-id | forward primer (5‘ – 3‘) | reverse primer (5‘ – 3‘) | Amplicon (bp) |
| --- | --- | --- | --- | --- |
|  |  |  |  |  |
| *fzd5* | NM_131134 | gattttggctgcatgtcctg | tcgtgattgaactggttggg | 148 |
| *fzda8a* | NM_130918 | tgcctttgtcccaagtctg | cgtaattgtttctgcatcgctg | 150 |
| *fzda8b* | NM_131553 | cgagcgggtcagtttacag | acgttctgcttgtcctgatg | 133 |
| *wnt8b* | NM_130959 | agtgataacgtgggatttggag | tcctctgcatggttcctttc | 139 |
| *wnt11* | NM_131076 | gatgccccaatgaagatgaag | aggaaccagaaacaccgtg | 139 |

Supplementary Table 5: qRT-PCR primer for the analysis of opsin/rhodopsin genes.

| Gene | Gene-ID | forward primer (5‘ – 3‘) | reverse primer (5‘ – 3‘) | Amplicon (bp) |
| --- | --- | --- | --- | --- |
| *opn1lw2* | NM_001002443 | ccaacagcaataacacaaggg | gcgacaaccacaaagaacatc | 109 |
| *opn1mw1* | NM_131253 | ggctgtgtaatggagggattc | atggtttgcggagaatttgaag | 132 |
| *opn1mw2* | NM_182891 | gctttcgctggaacaattatgg | acaagagaccaaagagcaacc | 149 |
| *opn1sw1* | NM_131319 | tcattttctcctactcacagctc | cacaaaagagccaaccatcac | 131 |
| *opn1sw2* | NM_131192 | ggttcctttcagcaccattg | agaagccgaacaccattacc | 146 |
| *rho* | NM_131084 | agtcctgcccagacatctag | gtactgtgggtattcgtatggg | 150 |
| *rhol* | NM_001110031 | cagagggaccagacttttacg | tgtgggtattcataagggctg | 73 |
|  |  |  |  |  |
|  |  |  |  |  |
